# Genome-wide impact of hydrogen peroxide on maintenance DNA methylation in replicating cells

**DOI:** 10.1186/s13072-021-00388-6

**Published:** 2021-03-24

**Authors:** Annika R. Seddon, Yusmiati Liau, Paul E. Pace, Allison L. Miller, Andrew B. Das, Martin A. Kennedy, Mark B. Hampton, Aaron J. Stevens

**Affiliations:** Department of Pathology and Biomedical Science, University of Otago, PO Box 4345, Christchurch, 8140 New Zealand

**Keywords:** DNA methylation, DNA methyltransferase, Epigenetics, Oxidative stress, Hydrogen peroxide, Cancer, Inflammation, Redox signalling

## Abstract

**Background:**

Environmental factors, such as oxidative stress, have the potential to modify the epigenetic landscape of cells. We have previously shown that DNA methyltransferase (DNMT) activity can be inhibited by sublethal doses of hydrogen peroxide (H_2_O_2_). However, site-specific changes in DNA methylation and the reversibility of any changes have not been explored. Using bead chip array technology, differential methylation was assessed in Jurkat T-lymphoma cells following exposure to H_2_O_2_.

**Results:**

Sublethal H_2_O_2_ exposure was associated with an initial genome-wide decrease in DNA methylation in replicating cells, which was largely corrected 72 h later. However, some alterations were conserved through subsequent cycles of cell division. Significant changes to the variability of DNA methylation were also observed both globally and at the site-specific level.

**Conclusions:**

This research indicates that increased exposure to H_2_O_2_ can result in long-term alterations to DNA methylation patterns, providing a mechanism for environmental factors to have prolonged impact on gene expression.

**Supplementary Information:**

The online version contains supplementary material available at 10.1186/s13072-021-00388-6.

## Background

Epigenetic modification of chromatin provides a mechanistic basis through which environmental stimuli can modulate gene expression. Epigenetic marks, such as methylation of cytosine and histone modifications, are utilized during development to generate a wide range of cellular phenotypes from the same DNA code. While the majority of epigenetic changes in adult organisms are due to the continued differentiation of stem cells, they can also be modified during ageing [1, 2] and by interactions with environmental stimuli such as cigarette smoke, pollutants, and alcohol consumption [3–9]. However, there is a lack of mechanistic studies that investigate how environmental factors impact DNA methylation.

Several potential environmental modifiers are associated with increased oxidative stress, including aging and chronic inflammation [10, 11]. Mitochondrial dysfunction is linked to increased superoxide and hydrogen peroxide (H_2_O_2_) production in a range of pathologies [12]. Ageing, smoking, radiation and xenobiotic metabolism can also result in increased H_2_O_2_ in biological systems [13–19]. Immune cells such as neutrophils and macrophages generate a range of reactive oxygen species following NADPH oxidase (NOX) activation, many of which are cell permeable, and contribute to oxidative stress during inflammation [11, 20, 21]. H_2_O_2_ and myeloperoxidase-derived oxidants produced by immune cells can oxidize redox-sensitive thiol proteins and methionine [22–24]. Importantly, methionine is a precursor of the methyl donor S-adenosyl-methionine (SAM).

We have previously reported that H_2_O_2_ and chloramines generated by phagocytic immune cells are able to inhibit DNA methyltransferase (DNMT) activity during DNA replication [25]. These results suggested that this inhibition was due to the oxidation of DNMT1’s catalytic site cysteine and occurred at concentrations that did not have major impacts on cell proliferation. DNMTs copy DNA methylation to the nascent DNA strand during DNA replication, a process which ensures that the correct pattern of methylation is maintained throughout cellular division. However, in a cell culture system, only the chloramines had a significant impact on global DNA methylation because they also depleted SAM [25].

In this study, we have revisited the impact of H_2_O_2_ on maintenance DNA methylation in cultured cells by using a more sensitive genomic approach that individually monitors 850,000 CpG sites across the human genome. We have also investigated the reversible nature of any modifications analysing changes seen at early and late timepoints. We reveal that sublethal doses of H_2_O_2_ caused a significant disturbance to DNA methylation patterns, increasing overall variability and site-specific methylation in the 1–2 h immediately following exposure to this oxidant. Many of the changes were lost 72 h after exposure, but not all. Collectively, these findings describe a potential mechanism for various environmental factors and pathologies that promote oxidative stress to impact the methylation pattern of proliferating cells.

## Results

**Fig. 1:**
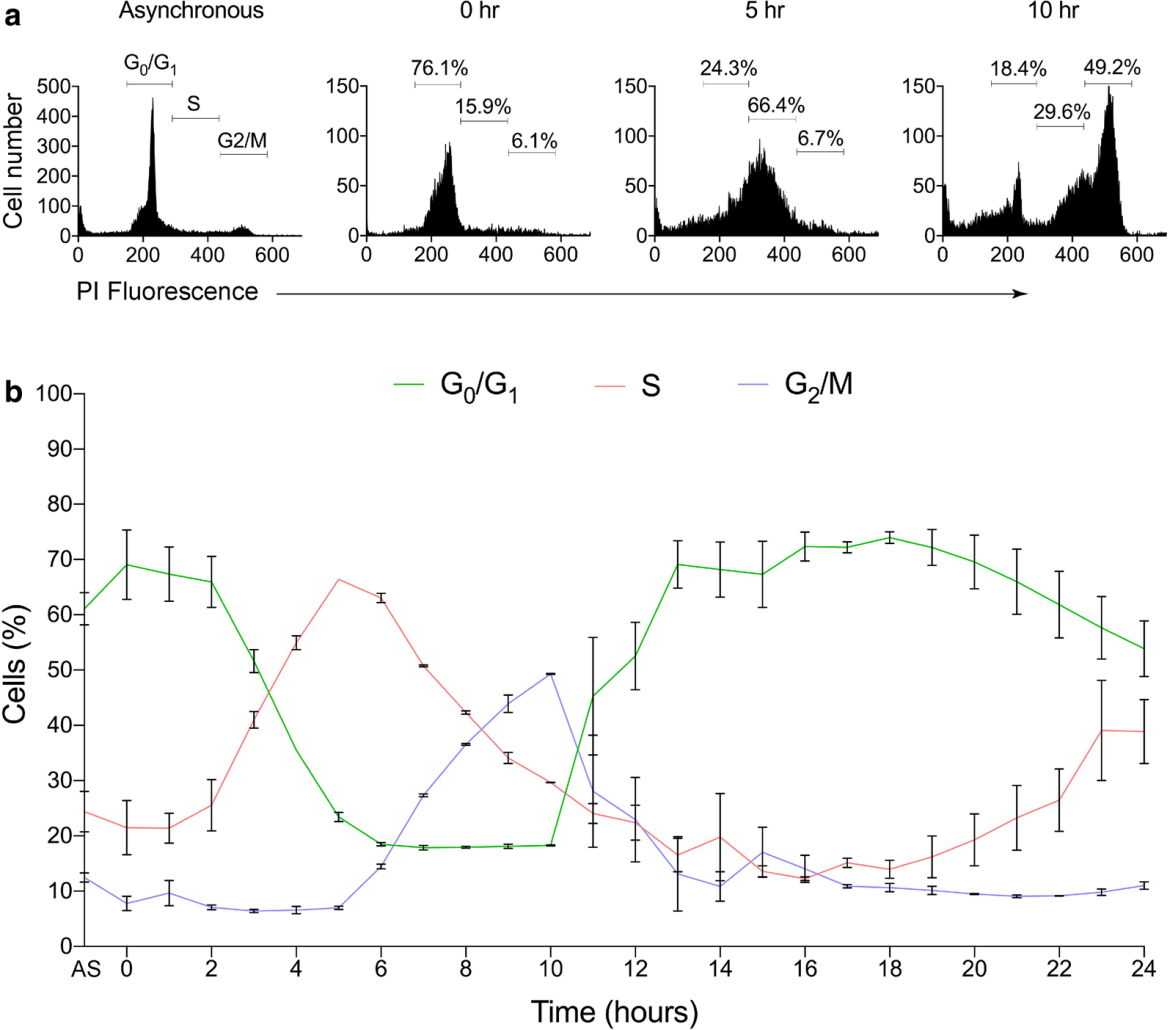
Cell cycle analysis 24-h post-release. **a** Representative flow cytometry histogram of the Jurkat cell cycle with percentage of cells on the y-axis and the mean fluorescence from PI staining (DNA content) on the x-axis. The percentage of cells in each phase of the cell cycle is shown for asynchronous cells, 0, 5 and 10 h post-release from thymidine block. AS on the x-axis, represents the cells in an asynchronous state. **b** Jurkat cell cycle analyses over 24-h post-release from thymidine block. Hours post-release are represented on the x-axis and DNA content, as cell percentages at G_0_-G_1_ (green), G_2_-M (blue) and S-phase (red) are represented on the y-axis. Error bars represent standard deviation of 2–3 technical replicates. DNA content was assessed using PI staining, followed by flow cytometry analyses

We propose that oxidative stress is able to inhibit the maintenance methylation that occurs following DNA replication in proliferating cells. Cultured Jurkat T-lymphoma cells were synchronized with a thymidine block to maximize the number undergoing DNA synthesis at the time of exposure to oxidative stress. Flow cytometry was used to monitor cell cycle transitions and determine the period of maximal DNA replication during the S-phase (Fig. 1a). The asynchronous population of Jurkat cells shows that just before cell division the majority of cells were in the G_0_/G_1_ phase, ~ 20% were in S-phase and ~ 14% of cells were in G_2_/M phase. The period of 2–5 h post-release was when the greatest number of cells entered the S-phase (Fig. 1b), and the time we chose to introduce H_2_O_2_.

**Fig. 2:**
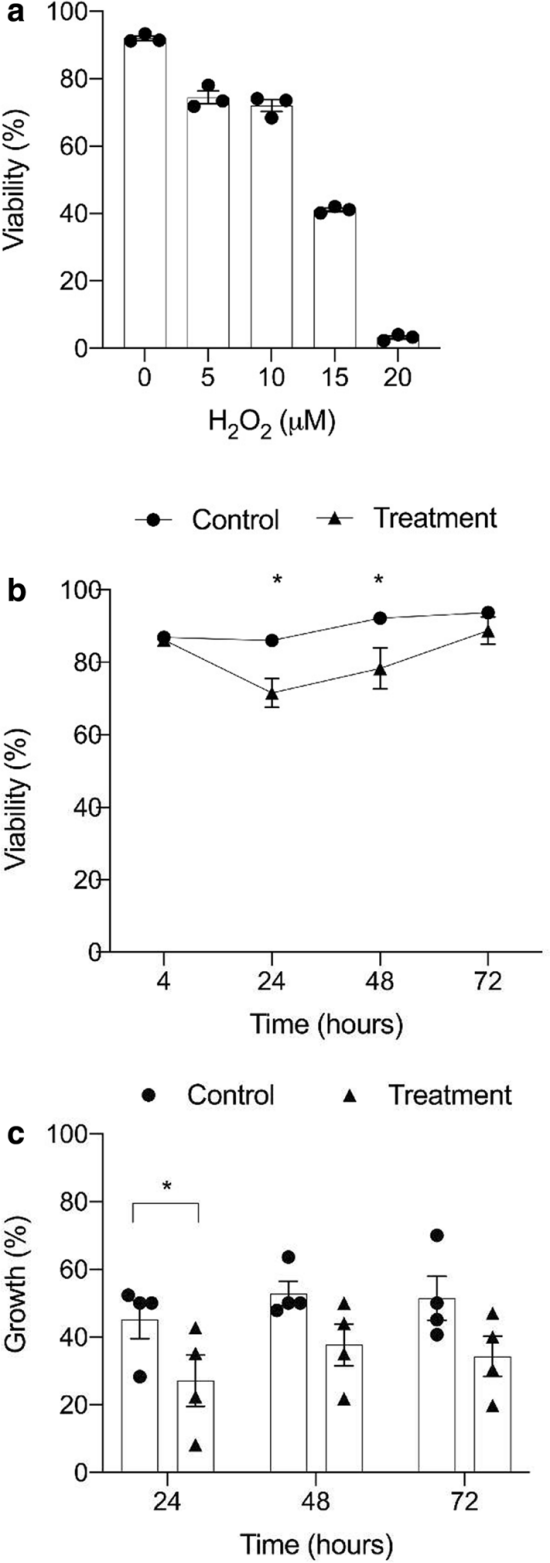
Influence of hydrogen peroxide exposure on cell viability and percentage growth of synchronized cells. **a** Cells were exposed to two doses of H_2_O_2_, at both 2 and 3-h post-release. Each dose was at the stated concentration on the x-axis and viability was assessed after 24 h. Data are means and SE of three independent experiments. **b **Viability was assessed by flow cytometry based on the exclusion of PI at 4, 24, 48 and 72 h post-release. **c **The percentage growth (y-axis) for each replicate at 24, 48 and 72-h post-release (x-axis). Live cell counts conducted at 24, 48 and 72-h post-release were compared to the previous day's counts and the percentage growth calculated ((current day count—previous day count)/previous day count × 100). Circles represent control samples and triangles represent treatment samples. Data are means and SE of four independent experiments. Significant differences were determined with paired t-tests and are denoted with asterisks * = *p* < 0.05

**Fig. 3:**
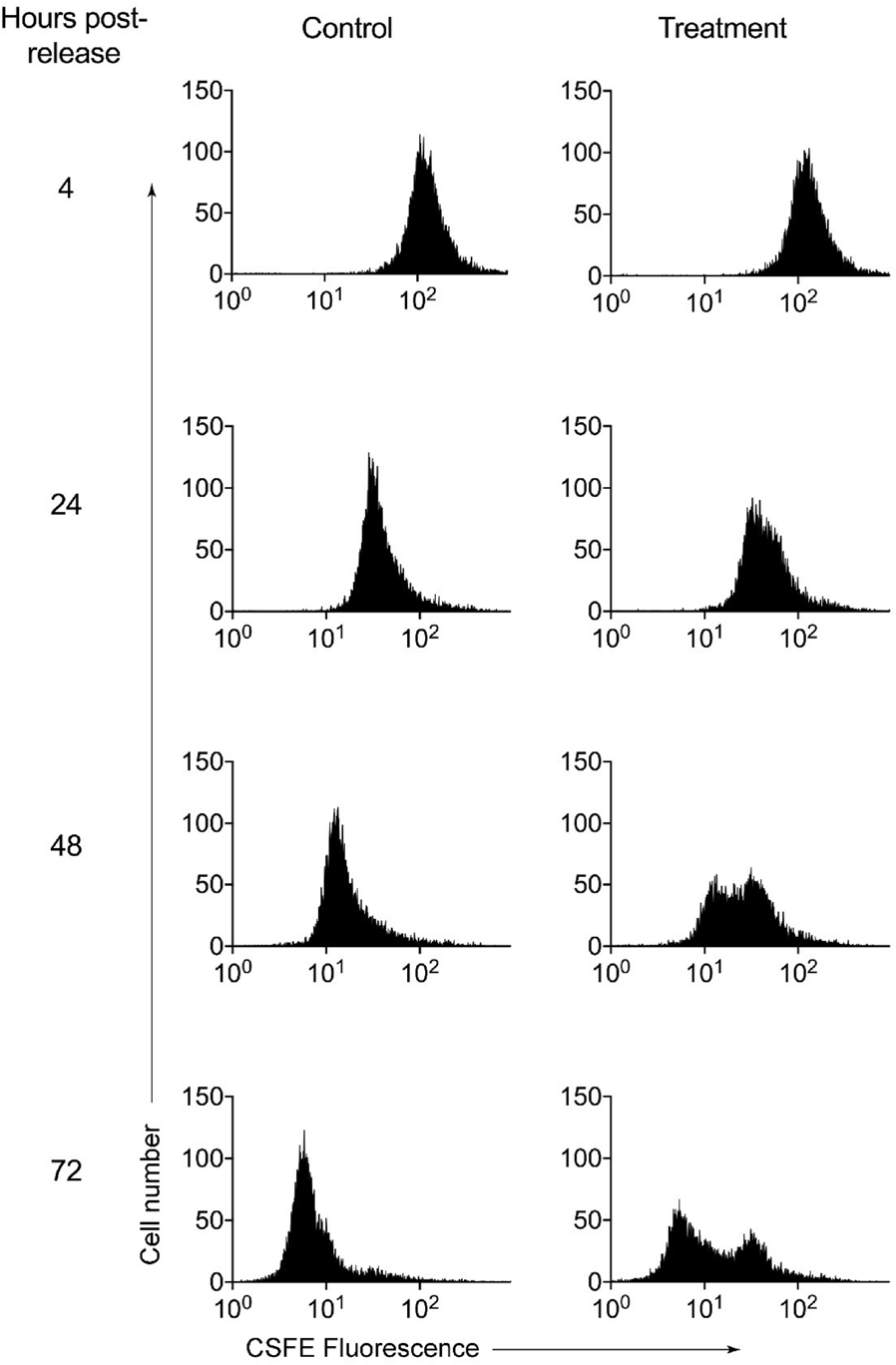
CFSE dilution of H2O2 treated cells at 24, 48 and 72-h post-release. Cell proliferation was observed by labelling cells with the cell permeable fluorescent dye CFSE and monitoring its dilution over 72 h for control and treatment. At each cell division, the fluorescence is roughly halved, enabling the visualization of the proliferation of labelled cell populations over time. The number of cells is displayed on the y-axis and the concentration of CFSE is on the x-axis. Graph shows the results from a representative experiment

H_2_O_2_ is consumed by Jurkat cells and media constituents within ~ 30 min of exposure [26, 27]. Cells were therefore exposed to two doses of H_2_O_2_, at 2 and 3 h post-release, to maximize the potential for inhibition of maintenance methylation during DNA replication. To determine the highest concentration of H_2_O_2_ that could be used without significant cell death, synchronized cells were exposed to a range of concentrations (Fig. 2a). Treatment with 15 µM H_2_O_2_ resulted in a large decrease in viability after 24 h, while 5 µM and 10 µM H_2_O_2_ treatments only had a small effect. With 10 µM H_2_O_2_, which was selected for subsequent experiments, there was no significant difference in viability between the treatment and control groups at 4 or 72 h (*p* = 0.25) (Fig. 2b). Assessment of cell growth indicated that only the control cells doubled in cell density after 24 h, whereas the H_2_O_2_-treated cells had significantly lower percentage growth (*p* = 0.02) (Fig. 2c). Cells were diluted to a concentration of 0.25 × 10^6^ cells/ mL at 24 h, and the decrease in fold growth for the treatment samples compared to control was still observed at 48 and 72 h, although not statistically significant (*p* = 0.1 and 0.6). This slowing of growth rate was confirmed by the analysis of CFSE dilution by flow cytometry (Fig. 3). Collectively this data shows that both control and treated cells underwent at least one round of division by the 72-h time point.

### DNA methylation analyses

DNA methylation profiles were assessed using the Illumina Infinium MethylationEPIC 850K array at 4 h and 72 h after the addition of cytidine to release cells from thymidine block, which was 2 and 70 h from the first H_2_O_2_ exposure, respectively. This technology assesses DNA methylation at over 850,000 CpG sites across the human genome, with nucleotide-level resolution.

### Principal component analysis (PCA) of DNA methylation and hierarchical clustering

**Fig. 4:**
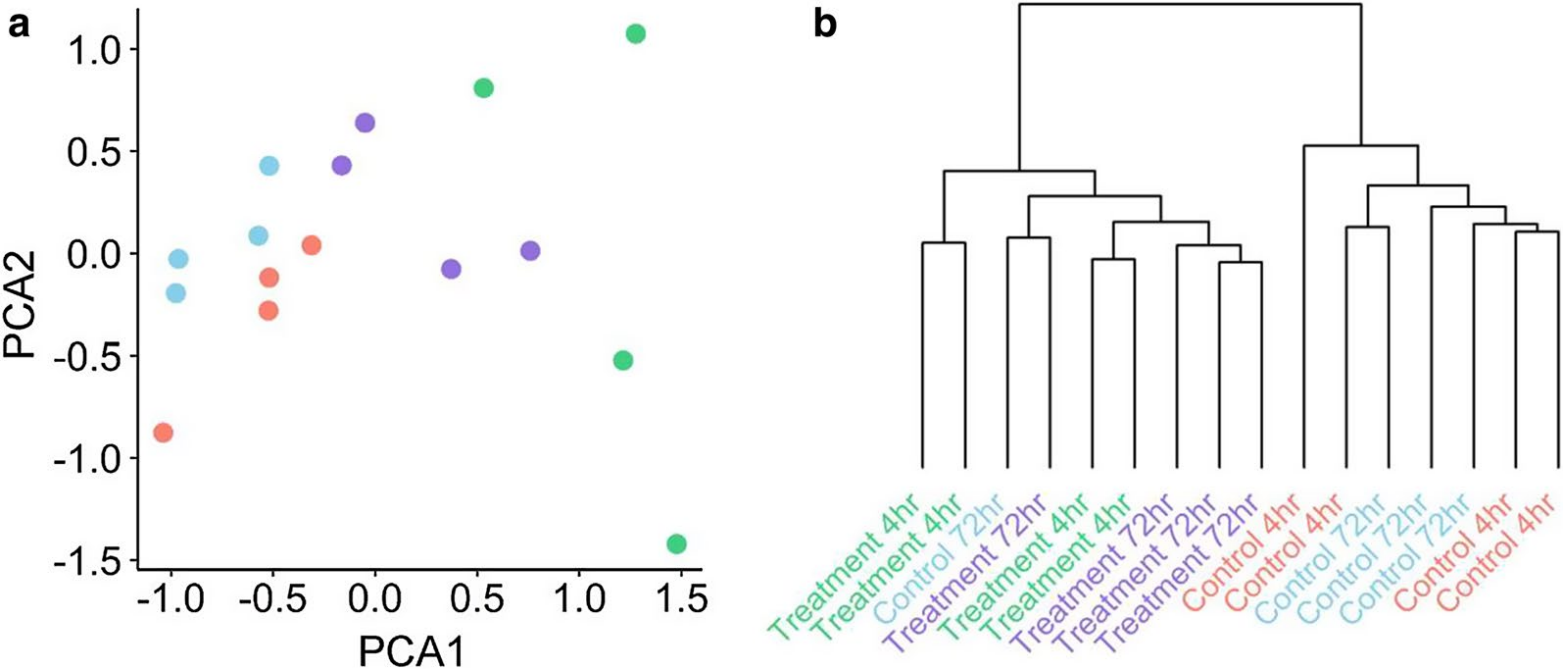
Unsupervised assessment of data variability. **a** Multidimensional scaling of M-values, with the distances for leading log_2_FC in dimension 1 represented on the x-axis and the leading log_2_FC in dimension 2 are represented on the y-axis. Red and blue dots represent control samples (4-h and 72-h time points respectively), green and purple dots represent H_2_O_2_ treated samples (4-h and 72-h time points, respectively). **b **Hierarchical clustering of β-values for all probes. The relative change in β-values is represented on the y-axis and individual samples are represented on the x-axis

Multidimensional scaling was used to assess the top contributing factors in data variability, which corresponded with treatment, and to a lesser extent time (Fig. 4a). Overall, there was a distinct separation between samples from the control and H_2_O_2_ treatment groups. Samples from the control group formed a relatively tight cluster with less spatial separation, and as expected there was no separation between time points for the control samples. The H_2_O_2_ treatment samples from the 72-h time point more closely represented the control samples than the H_2_O_2_ treatment samples from control at the 4-h time point.

Hierarchical clustering was performed on a distance matrix calculated using the β-values for all probes, regardless of significance. Except for one sample, which incorrectly grouped within the treatment, this approach successfully differentiated between the control and H_2_O_2_ treatment groups (Fig. 4b).

### Relative average DNA methylation change

**Fig. 5:**
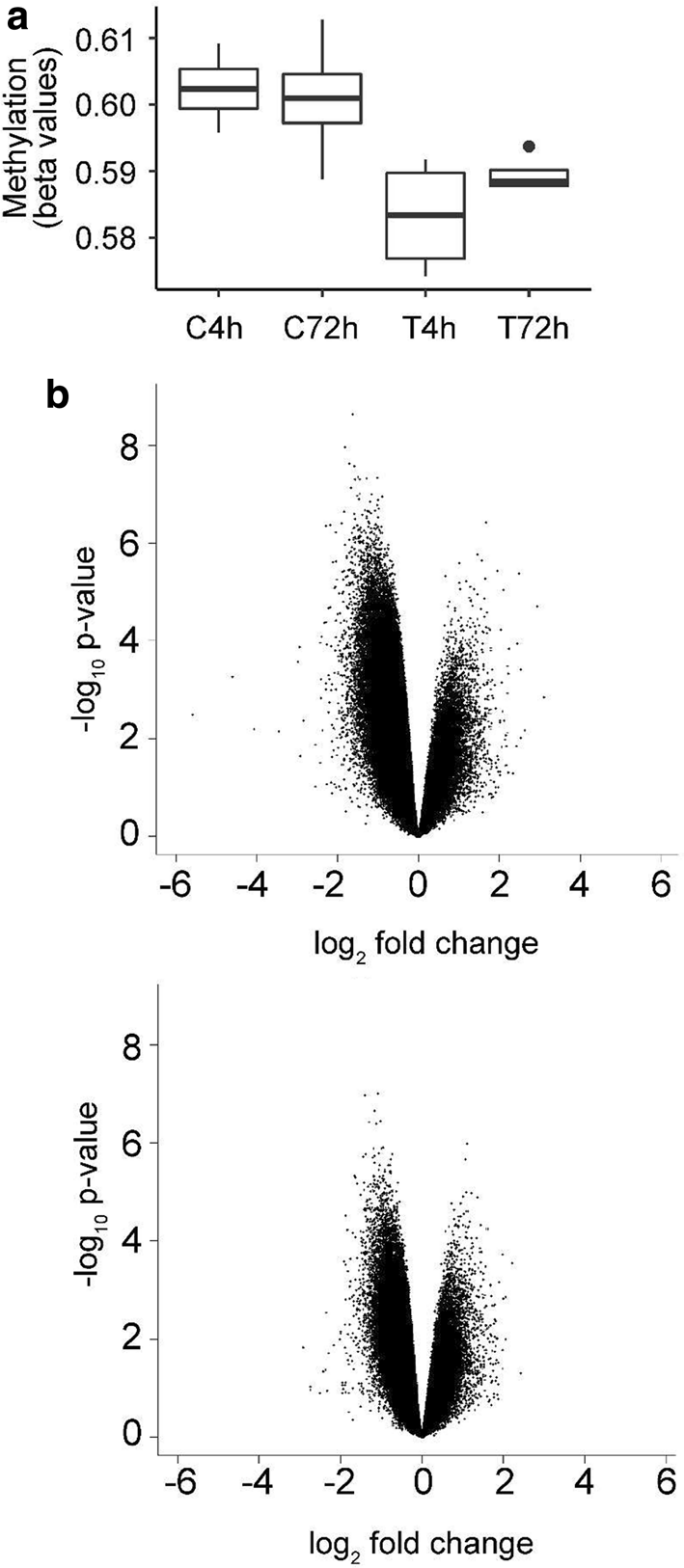
Analysis of overall DNA methylation change. **a** Relative average methylation levels for each group. Mean methylation levels for β-values are presented as percentages for each treatment (T) and control (C) group. **b** Volcano plots displaying log_2_-fold changes versus a measure of statistical significance (log_10_ *p*-value) for the 4-h time point (upper panel) and the 72-h time point (lower panel)

At the 4-h time point, the mean methylation level of the H_2_O_2_ treatment group was 3.33% less than the control group (*p* = 0.013, t_*(5)*_ = 3.72) (Fig. 5a). However, at 72 h this significant difference was no longer observed (*p* = 0.10, t_*(4)*_ = 2.2). There was no significant difference between the 4-h and 72-h time points within each group, although the H_2_O_2_ treatment group demonstrated a minor increase and had substantially reduced variability (Fig. 5a).

Plotting the individual probe-wise comparisons of significance versus effect size change confirmed there was a substantial decrease in methylation after H_2_O_2_ treatment_,_ with several probes from the 4-h time point demonstrating a significant log_2_FC greater than  ~ 1. There was a comparative reduction in the effect size and significance at the 72-h time point, which indicates that the H_2_O_2_ treatment and control samples were more similar to each other than at the 4-h time point (Fig. 5b, c). Together these observations suggested that on a global scale, there were significant decreases in DNA methylation in the treatment group compared to the control group at 4 h, which were largely restored by 72 h (Fig. 5).

### Analysis of variability in DNA methylation after H_2_O_2_ treatment

**Fig. 6:**
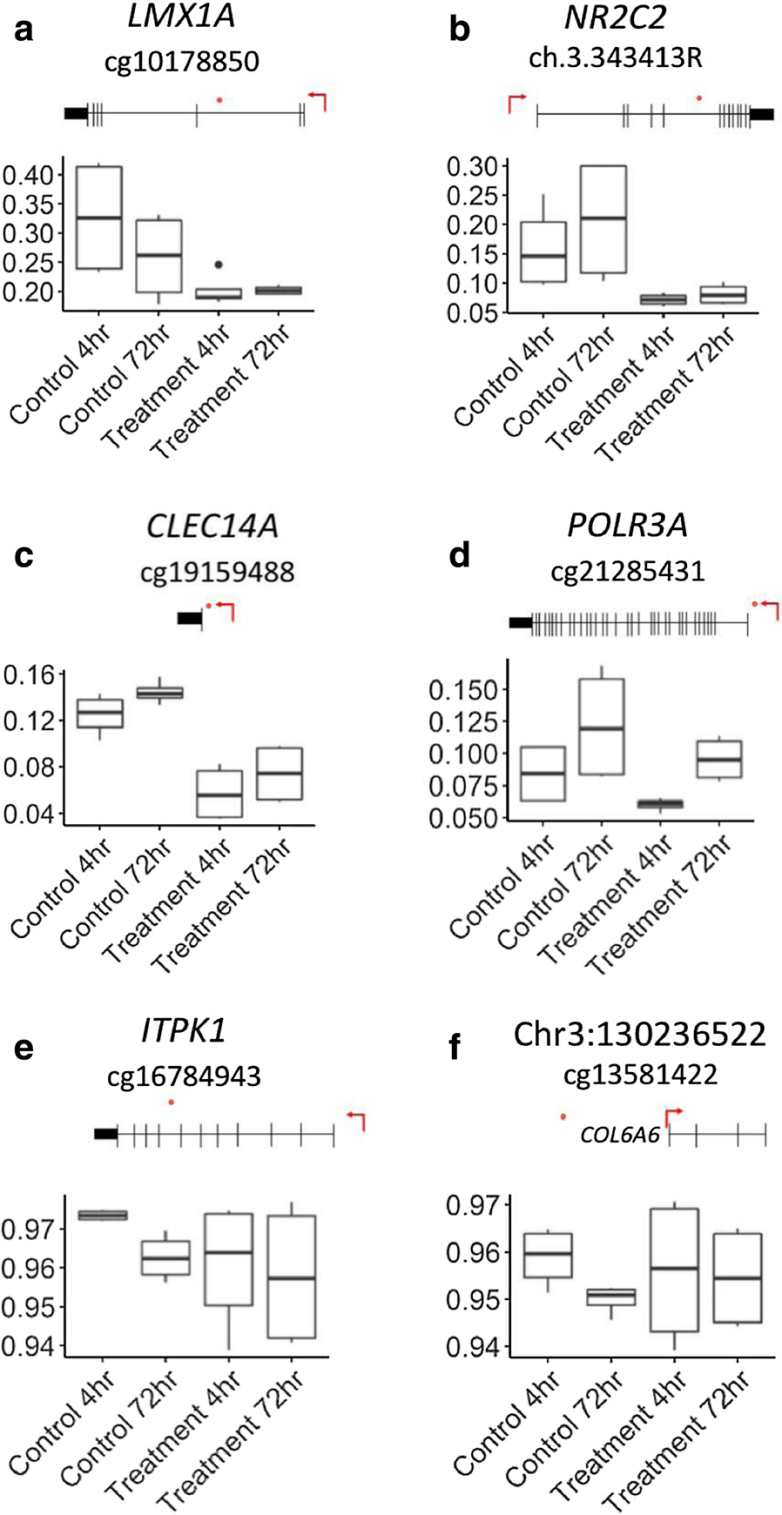
Box plots showing representative β-values of a subset of the top differentially variable probes**.** Probes were selected that demonstrated a consistent effect at both the 4-h and 72-h time points. β-values are represented on the y-axis and group on the x-axis. The CpG sites are marked with an orange dot in the representative scheme placed above each graph. The transcriptional start site is marked by a red arrow: **a** chr1:165,265,507; **b** chr3:15,058,168; **c** chr14:38,727,024; **d** chr10:79,789,707; **e** chr14:93,415,143; **f** chr3:130,236,522

Principal component analysis and global methylation change suggested that there was a higher level of heterogeneity in the DNA methylation profiles after H_2_O_2_ treatment. To investigate the individual probes that contributed towards this observation, we assessed the differential variability at individual CpG positions (DVPs) using a general linear model that accounted for biological replicates. At the 4-h time point, 1035 DVPs demonstrated a significant increase in variability in the H_2_O_2_ treatment group compared to the control, and 286 demonstrated a significant decrease in variability. At the 72-h time point only 41 DVPs demonstrated an increase in variability and 115 demonstrated a decrease. Forty-six DVPs were common between the two time points, however, only 18% (7 DVPs) showed a consistent direction of change (Fig. 6).

### Differentially methylated CpGs at the 4-h time point

**Fig. 7:**
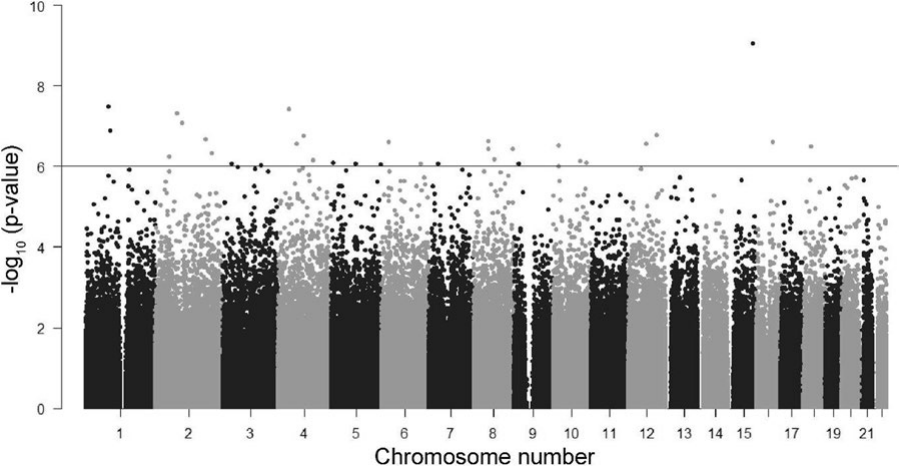
Manhattan plot of all probes (M-Values) in the 4-h time point. All probes are ordered per chromosome position along the x-axis, and *p*-values as the –log_10_(*p*-values) are presented on the y-axis. Genome-wide significance was determined using the “Benjamini, Hochberg” method within Limma is represented by the horizontal black line

**Fig. 8:**
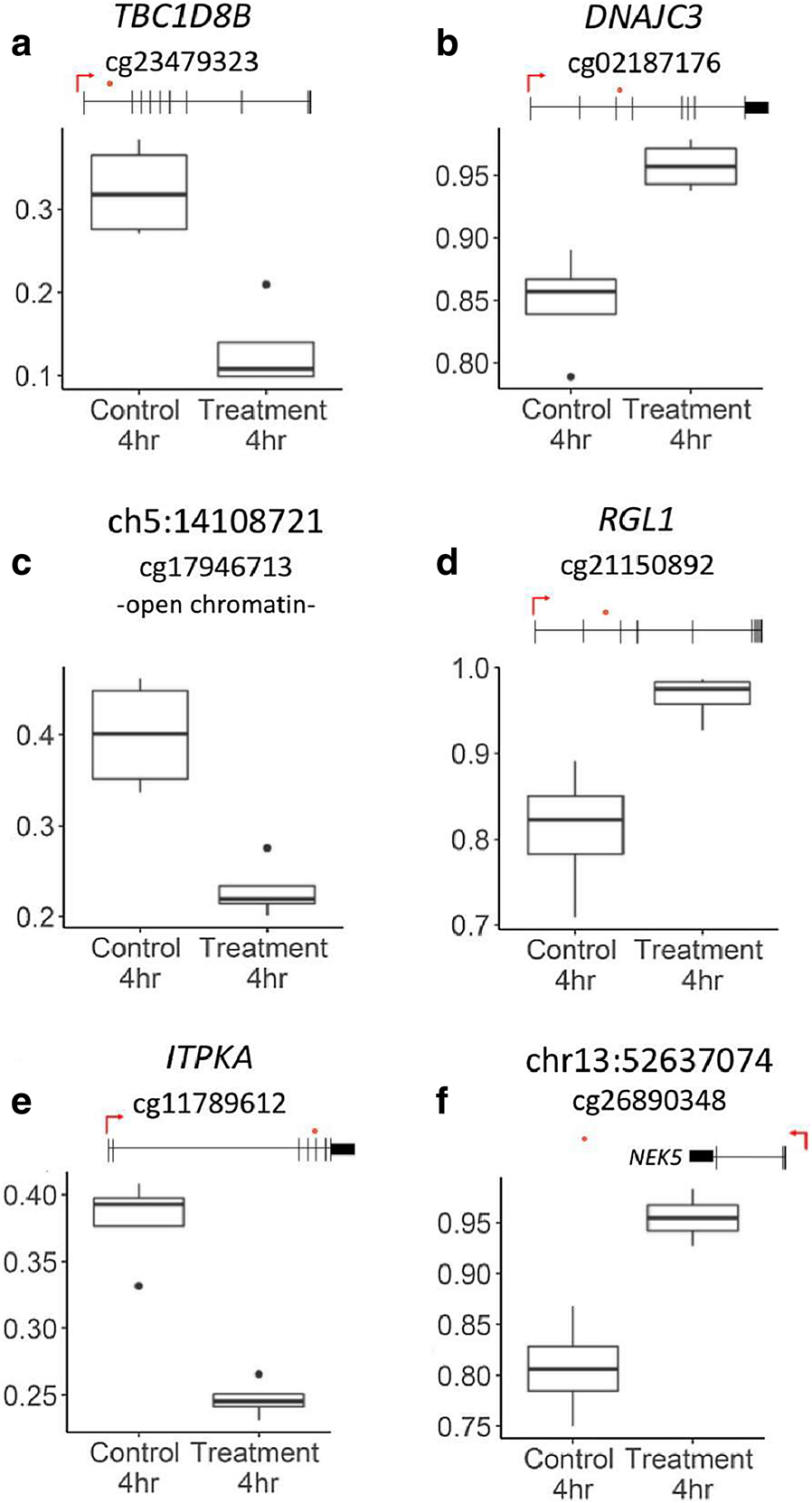
Box plots showing representative β-values at the 4-h time point for selected probes with a log_2_FC > 1.2. β-values are represented on the y-axis and group on the x-axis. The CpG sites are marked with an orange dot in the gene scheme placed on top of each graph, with the TSS is marked by a red arrow: **a** chr15:41,794,412; **b** chr13:96,367,153; **c** chr5:14,108,721; **d** chr1:183,846,009; **e** chrX:106,061,616; **f** chr13:52,637,074

To identify changes that associated with H_2_O_2_ treatment we assessed DNA methylation levels at each individual probe using the “topTreat” algorithm, within the R package, Limma. To summarize the results, each probe was mapped to the corresponding genomic region and unadjusted significance was plotted across the whole genome as a Manhattan plot (Fig. 7). This analysis demonstrated that the significant changes were distributed across the genome, and did not appear to be concentrated at specific genomic locations. There were 1162 probes that demonstrated a significant decrease in methylation using a log_2_FC (M-values) cutoff of 1, and 89 probes that demonstrated a significant increase in methylation (adj. *p* < 0.05). Increasing the log_2_FC cutoff to 1.2 identified 87 probes that demonstrated a significant decrease in methylation and three probes that demonstrated a significant increase (Additional file 1: Table S1). The top most significant probes had an initial methylation value of 20–25% in the control group, and a value of 10–15% in the H_2_O_2_ treatment group (Fig. 8a). Therefore, the raw data for the top six most significant probes that did not conform to this general trend are presented in Fig. 8 (including the three probes that demonstrated increased methylation). The top 20 significantly differentially methylated positions with a log_2_FC > 1.2 in order of largest fold change are presented in Table 1.

**Table 1:** The top 20 most significant differentially methylated genes with a log_2_FC > 1.2 at the 4-h time point, ordered by magnitude of log_2_-fold change

Probe ID	log_2_FC (4 h)	Adj *p-*val (4 h)	log_2_FC (72 h)	Chromosome	Location	Gene
cg21150892	2.94	0.04	0.27	1	183,846,009	RGL1
cg26890348	2.48	0.03	0.87	13	52,637,074	
cg24024260	− 2.29	0.01	− 0.40	2	182,174,340	LOC101927156
cg05047191	− 2.23	0.04	− 0.33	12	15,304,108	RERG
cg00192946	− 2.19	0.01	− 0.95	X	32,430,274	DMD
cg21426441	− 2.19	0.04	− 0.77	X	17,260,259	
cg14954143	− 2.13	0.02	− 0.79	3	162,071,211	
cg02187176	2.1	0.04	0.61	13	96,367,153	DNAJC3
cg23727674	− 2.09	0.03	− 0.30	2	148,602,993	ACVR2A
cg25969765	− 2.07	0.03	− 0.80	18	58,350,853	
cg11372135	− 2.05	0.01	− 0.54	10	18,629,284	CACNB2
ch.X.16400295F	− 2.04	0.02	− 0.76	X	16,490,374	
cg03012782	− 1.87	0.03	− 1.08	3	112,250,543	
cg06780892	− 1.87	0.04	− 0.77	7	20,638,177	
cg15074165	− 1.85	0.03	− 1.00	X	71,792,745	HDAC8
cg09333215	− 1.84	0.01	− 0.17	1	88,475,866	
cg01492091	− 1.81	0.02	− 0.91	7	31,460,998	
cg08892255	− 1.8	0.03	− 1.02	20	21,928,217	
cg07042371	− 1.77	0.02	− 0.71	5	54,468,233	MIR449C
cg01769501	− 1.77	0.04	− 0.67	4	76,912,251	SDAD1

log_2_FC, log_2_-fold change of M values

Adj.*p*-val, adjusted *p*-value

### Differentially methylated CpGs at the 72-h time point

At the 72-h time point, 19 probes demonstrated a significant decrease in methylation and one probe demonstrated a significant increase in methylation (adj. *p* < 0.05), when assessed using a log_2_FC cutoff of 1 (Additional file 1: Table S2). The magnitude of these changes was less than 10%, therefore there were no significant probes detected at a log_2_FC cutoff of 1.2 at the 72-h time point.

**Fig. 9:**
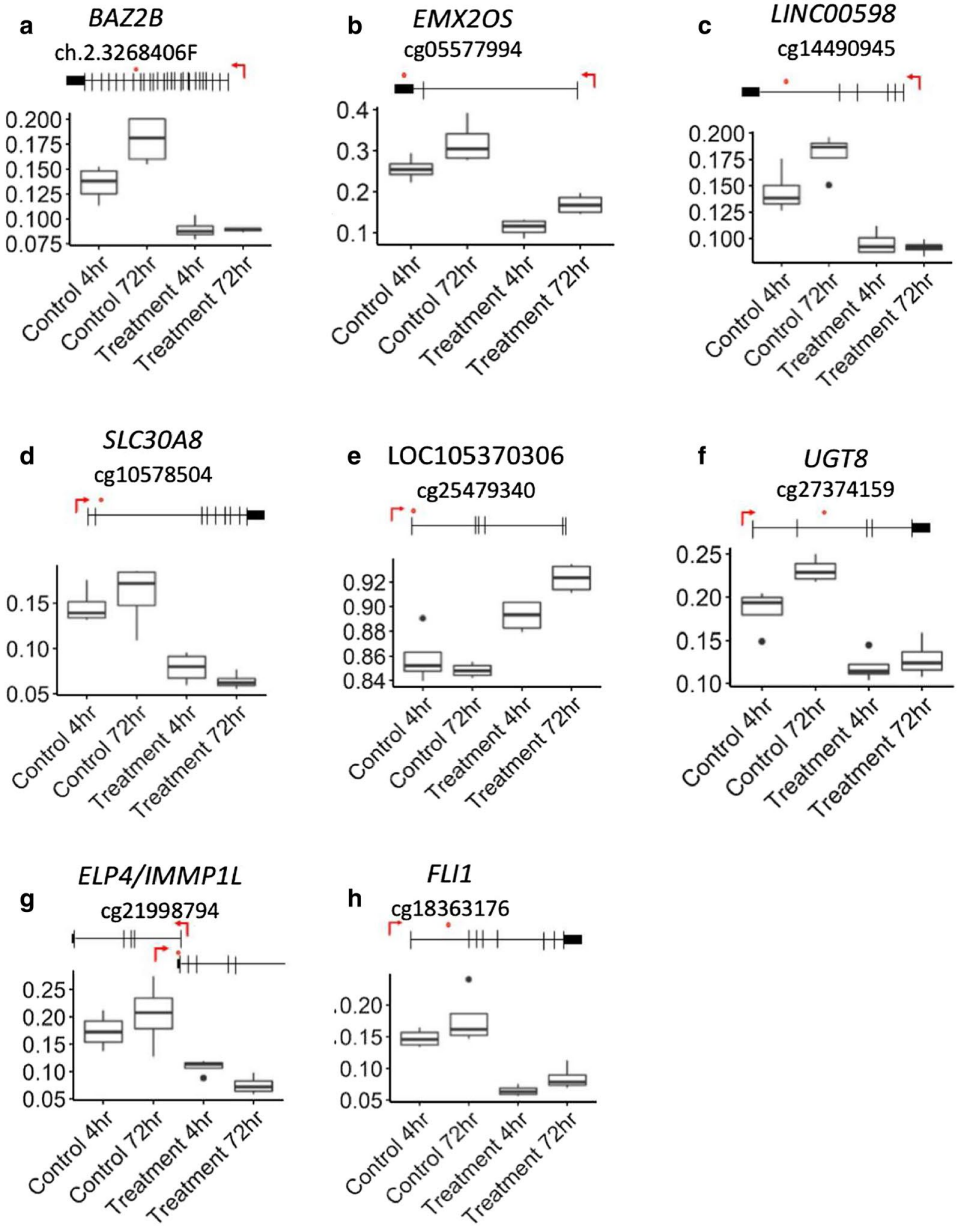
Box plots showing representative β-values at the 72-h time point. β-values are represented on the y-axis and group on the x-axis. The CpG sites are marked with an orange dot in the scheme placed above each graph, with the TSS is marked by a red arrow. Antisense non-coding RNA is indicated in grey. **a** chr2:160,224,706; **b** chr10:119,254,968; **c **chr103:40,996,323; **d** chr8:118,145,984; **e **chr13:88,794,067; **f **chr4:115,580,778; **g **chr11: 31,530,551; **h **chr11: 128,606,110

Seventeen of the 20 significant probes detected at the 72-h time point were also detected in the 4-h time point when a log_2_FC of > 1 was used (Table 2) and the raw data from a subset of these probes are presented in Fig. 9. Two probes were also detected in the 4-h time point when a log_2_FC > 1.2 was used (cg05577994 mapping to gene: *EMX2OS* at chr10:119,254,968, adj. *p* < 0.05 and cg18363176 mapping to gene: *FLI1* at chr11:128,606,110, adj. *p* < 0.04). Both probes had a smaller log_2_FC at 72 h than at 4 h. However, all other significant probes showed a substantially larger log_2_FC at 72 h than at 4 h (Table 2).

**Table 2:** Top 20 differentially methylated probes with a logFC > 1 for the 72-h time point, ordered by magnitude of log_2_-fold change

Probe ID	log_2_FC (4 hr)	log_2_FC (72 hr)	Adj *p*-val (72 h)	Gene	Chromosome	Location
cg21998794	− 0.78	− 1.72	0.04	ELP4;IMMP1L*	11	31,530,551
cg02947434	− 0.62	− 1.72	0.05		12	33,728,505
cg26627970	− 0.45	− 1.64	0.04	KHDRBS2	6	62,993,589
cg13484357	− 1.28	− 1.6	0.05	SLC30A8*	8	118,125,096
cg10578504	− 1.02	− 1.54	0.04	SLC30A8*	8	118,145,984
cg06328725	− 1.09	− 1.5	0.05	PACRG*	6	163,171,959
cg05620710	− 0.71	− 1.48	0.05	*	5	140,010,064
cg05403127	− 1.04	− 1.43	0.06	LINC00703*	10	4,425,587
cg05655613	− 0.59	− 1.42	0.02	*	11	105,101,601
cg08223309	− 0.98	− 1.41	0.04	*	4	14,390,632
cg01909661	− 0.73	− 1.4	0.05	PTCHD1-AS*	X	22,446,176
cg11932468	− 1.28	− 1.38	0.05	TRPA1;MSC-AS1*	8	72,954,594
cg19281347	− 1.09	− 1.38	0.05	*	2	83,883,578
cg02153561	− 0.75	− 1.37	0.06	*	18	70,077,275
cg06951009	− 1.19	− 1.35	0.06	*	4	12,547,176
cg22181263	− 0.23	− 1.31	0.02		6	63,922,844
cg21898358	− 1.01	− 1.31	0.06	LILRA*	19	54,805,375
cg18363176	− 1.32	− 1.26	0.05	FLI1**	11	128,606,110
cg05577994	− 1.45	− 1.25	0.04	EMX2OS**	10	119,254,968
cg23537032	− 0.61	− 1.25	0.06	*	6	24,165,998

*Probes displayed a significant adj *p*-value at 4 h (log_2_FC > 1)

**Probes displayed a significant adj *p*-value at 4 h (log_2_FC > 1.2)

log_2_FC, log_2_-fold change of M values

Adj.*p*-val., adjusted *p*-value

### Top differentially methylated gene regions

**Fig. 10:**
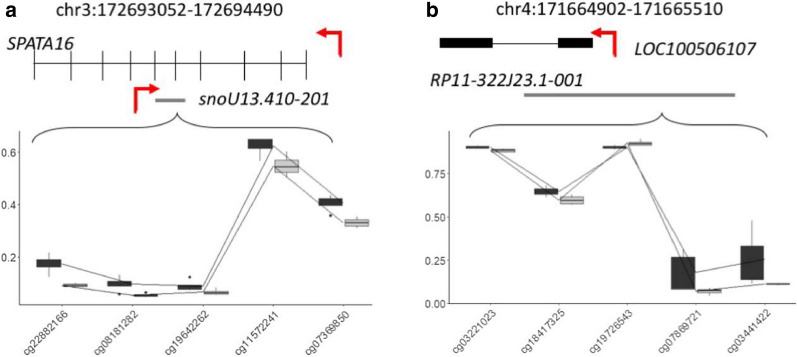
Differentially methylated gene regions. DMRs that displayed a significant change in methylation (y-axis represents β-values) across five CpGs (x-axis) between the treatment (grey) and control (black) for both 4-h and 72-h time points. Gene structure is placed on top of each graph, with the TSS is marked by a red arrow, grey lines represent non-coding RNA: **a** chr3:172,693,052–172,694,490, width:1439 bp; **b **chr4:171,664,902–171,665,510, width: 609 bp

We next investigated if multiple probes mapping to the same genomic loci demonstrated consistent methylation changes. We limited this analysis to differentially methylated regions (DMRs) that contained five or more adjacent significant CpGs, with an average change in methylation larger than 5%. Under these parameters, we detected 70 significant DMRs at the 4-h time point and five significant DMRs at the 72-h time point (Additional file 1: Tables S3 and S4). Two significant DMRs were consistent between the 4-h and 72-h time points (Table 3 and Fig. 10).

**Table 3:** Significant DMRs that were observed at both the 4-h and 72-h time points

Promoter	4 h			72 h		
	FDR	Max β-values FC	Mean β-values FC	FDR	Max β-values FC	Mean β-values FC
snoU13.410–201	8 × 10^–5^	− 0.1	− 0.05	5 × 10^–8^	− 0.08	− 0.05
RP11-322J23.1–001	0.012	− 0.1	− 0.05	2 × 10^–4^	− 0.1	-0.05

*FDR* false discovery rate, *FC* fold change

The genes corresponding to the top most significant CpG sites at 4 h, were assessed for significant biological relevance by pathway analysis within the GO and KEGG databases. Neither comparison identified significantly enriched pathways, after correction for multiple testing (Additional file 1: Table S5).

## Discussion

We used methylation arrays to investigate if exposure to H_2_O_2_ during DNA replication could interfere with the transfer of DNA methylation patterns in proliferating cells. H_2_O_2_ exposure during the S-phase of DNA replication disrupted DNA methylation of the nascent strand in proliferating cells 2 h after the first exposure to H_2_O_2_, with most but not all of these changes reversed by 72 h. By 72 h, all cells had undergone at least one round of division, indicating that these changes were representative of the epigenome of daughter cells. Sublethal H_2_O_2_ is known to alter gene expression in the short-term through impacting the activity of redox-sensitive transcription factors. Our results provide a mechanism by which H_2_O_2_ may have long-term impacts on gene expression and cell function.

Because aliquots of the treatment and control samples were split from the same source immediately prior to treatment, methylome changes relating to cell cycle were controlled for in the study design. The design of these experiments was such that DNA methylation was assessed 1–2 h after treatment, thus comparing the efficiency of nascent DNA methylation between the control and treatment samples. DNA methylation was then assessed again at 72 h to establish if the observed changes were corrected, or were inherited into the epigenome of subsequent cell generations. Cell viability and live cell counts indicated that approximately 72 h was required before cellular replication in the treatment group resembled that of the control group. This is likely to reflect the length of time required for repair of DNA damage, and re-establishment of the epigenome, and therefore DNA methylation was not assessed at the 24- and 48-h time points. An unsupervised assessment of data variability indicated that the treatment samples from the 4-h time point grouped distinctively from all other samples. There was no noticeable separation between the control samples, which more closely resembled the 72-h treatment samples.

Analysis of the average relative DNA methylation levels between groups indicated that H_2_O_2_ exposure corresponds with a significant decrease of ~ 3.3% in average methylation between treatment and control at the 4-h time point, however, there was no significant change between the 72-h time points. This change was driven by the combined effect of many probes with a log_2_FC approximately equal to one, however, at 72 h the effect sizes (and therefore significance) were substantially reduced. Hierarchical clustering was relatively successful at distinguishing the control samples from the treatment samples, and also identified that samples from the same biological replicate were more closely related, an effect which was subsequently controlled for in the statistical design.

These findings build on our previous work where we showed that while H_2_O_2_ inhibited DNMT, there was no detectable change in global methylation levels [25]. The main experimental difference our previous work was that H_2_O_2_ exposure occurred at the time of cytidine release. We modified our previous protocol by treating the cells twice with 10 µM H_2_O_2_ during the S-phase of DNA replication, at 2 and 3 h after the replication block was released, in order to maximize H_2_O_2_ exposure during the time period where DNMTs are most active [28]. Furthermore, while our previous study utilized a mass spectrometry approach which analysed the methylation of incorporated heavy-labelled deoxycytidine in the nascent strand, the current study used array technology that increased both the magnitude of the effect size and sensitivity, enabling detection of very small changes that were not previously possible using traditional methods. Our current work also highlighted the importance of nucleotide resolution, which enabled the detection of both increased and decreased methylation.

Intriguingly, there was a substantial increase in differential variability at both global and site-specific measurements. Differential variability of methylation at for the 4-h time point was significantly increased for the H_2_O_2_ treated samples compared to controls. Theoretically, his could either be due to increased variability at specific sites or random sites across the genome. We found that 1035 probes had significantly increased variability associated with H_2_O_2_ treatment at 4 h, which was reduced (41 probes) by 72 h. Therefore, site-specific increases in variability were contributing to the global variability in our system. It is important to note that there was also low consistency in the direction of change between the two time points, where 82% of the probes that were significant at both time points showed contrasting results.

The role of stochasticity in biological systems has received increasing attention in multiple contexts [29]. Whether cell-intrinsic or extrinsic, stochasticity can impact on a wide range of cellular processes including epigenetics and cell differentiation. Multicellular organisms have a remarkable capacity to produce consistent phenotypic outcomes even when challenged by variable conditions [30, 31]. This “buffering capacity” of normal cells is dysregulated in cancer and can be unmasked by treatment with a toxic stress such as chemotherapy. In practical terms, epigenetic heterogeneity in response to an environmental stress enables a range of transcriptional states across a population, some of which might confer resistance. This has been observed in acute myeloid leukaemia where epigenetic heterogeneity has been correlated with poorer outcomes and a shorter time to relapse [32]. Our finding that treatment with H_2_O_2_ increased the variability of methylation at the early time point is consistent with this and potentially points to a mechanism that allows a proportion of cancer cells across a population to adapt to oxidative stress.

We also investigated significant differentially methylated sites that demonstrated the largest fold change after H_2_O_2_ treatment. At the 4-h time point, 1162 probes showed a significant reduction in DNA methylation (log_2_FC > 1) in the treatment samples compared to control samples. In addition to this, 90 probes had an effect size larger than log_2_FC of 1.2, which suggests that in these instances the methylation level of the parental DNA strand may have been influenced [33]. With the exception of two probes (mapping to *EMX2OS and FLI1*), these 90 probes did not show a significant change at the 72-h time point. This suggests that the large changes observed at the 4-h time point were eventually corrected, or occurred in cells that eventually died. The majority of the significant probes at the 72-h time had a smaller effect size at 4 h (log_2_FC ~ 1), which suggests that they represent a delayed effect. Although no significant enrichment of gene pathways were observed, several of the top most differentially methylated genes are disrupted in pathologies such as diabetes and cancer [34–45].

Two gene regions corresponding to the *SPATA16* gene and *LOC100506102* (also overlapping with the small nuclear RNA: snoU13.410–201, and the miRNA: RP11-322J23.1–001) demonstrated significant differential methylation at both 4 h and 72 h. The potential results of these changes are unclear; however, the *SPATA16* gene is related with sperm count and motility. Extensive research has suggested that reactive oxygen species are a contributing factor to male infertility [46], with oxidative stress identified as a common pathology in approximately half of all infertile men [47]. Deregulation of snoRNA has been observed in several different cancer types including chronic lymphocytic leukaemia [48], hepatocellular carcinoma [49], colorectal cancer [50] and endometrial cancer [51], and also metabolic stress disorder [52]. Their roles as diagnostic and prognostic biomarkers have also been extensively studied [50, 53, 54].

Oxidants can directly damage and modify DNA, leading to mutations and genetic instability, but they can also cause interference in epigenetic pathways. One of the most common products of cellular oxidative stress is oxidized guanine or 8-hydroxy-2′-deoxyguanosine (8-OHdG) [55–57]. Increased frequencies of 8-OHdG in hepatocellular carcinoma patient DNA have been associated with increased oxidative stress, and in vitro studies have revealed that H_2_O_2_ exposure (50—250 µM) increased the occurrence of 8-OHdG and corresponded with altered methylation patterns in promoter regions of tumour suppressor genes [58]. When 8-OHdG is formed in close proximity to CpG sites, it has been shown to interfere with DNMT1 activity by impeding its access to the nascent strand, resulting in impaired maintenance methylation [59]. There is also evidence that 8-OHdG, through the recruitment of 8-oxoguanine DNA glycosylase-1, activates demethylation by ten-eleven translocation methylcytosine dioxygenase 1 (TET1) [60]. However, one study has suggested that the cumulative occurrence of 8-OHdG is low [61], and no reports have demonstrated specific enrichment at CpG sites. Therefore, 8-OHdG formation due to H_2_O_2_ exposure, particularly at low concentrations, would be unlikely to account for the large genome-wide decreases in methylation observed in our study. Another potential mechanism for regulation of methylation levels by oxidants is decreased TET activity. TET enzymes are sensitive to redox regulation, as their iron-dependent catalytic regions require ascorbate for optimal activity [62–65]. Oxidants such as H_2_O_2_, chloramines and superoxide, ubiquitous in an environment of oxidative stress, have been shown to deplete ascorbate levels, and could contribute to reduced TET activity [66, 67].

Alternatively, since there have been several reports that H_2_O_2_ can directly inhibit DNMT1 activity, it is more likely that the transient inhibition of DNMT1 due to H_2_O_2_ exposure is largely responsible for the decreased methylomic changes observed [25, 68]. However, the methylation changes from the 4-h time point were spread across the genome, with no strong evidence for enrichment of specific gene pathways, and yet sequence-specific effects on DNA methylation were observed across four independent experiments. This result would not be anticipated if H_2_O_2_ were acting through inhibition of DNMT1 activity alone. One possibility is that since many of the changes were consistent across multiple adjacent probes, the effects of H_2_O_2_ treatment relate to the positioning of DNMT1 at the time of exposure. The cells were released from synchronization at the same time, therefore they would progress through the cell cycle at a similar rate. This may indicate that there are undiscovered mechanisms that combine with inhibition of DNMT activity to yield sequence-specific alterations in DNA methylation.

Most of the large epigenetic changes reported in the literature are associated with long-term exposure to environmental stressors such as smoking and chronic inflammatory disease states [5, 69–72], it was therefore unexpected to observe a large number of methylation changes with a relatively short oxidant exposure period. However, our findings have a substantial effect size, and the oxidant concentration used in this analysis is similar to in vivo concentrations observed during chronic infection [73–76]. Although it is unlikely that a large population of cells would exist in S-phase at any one time point in vivo, in the situation of prolonged inflammation, a large proportion of cells will be exposed to a range of oxidants during cellular replication. Therefore, it is conceivable that H_2_O_2_-induced inhibition of DNMT could also occur in an asynchronous population of replicating cells in an inflammatory environment. Furthermore, although the current study was limited by the utilization of a single cell type, DNMT inhibition due to H_2_O_2_ treatment has been reported in other cell lines [68], and therefore methylation changes as a result of oxidative stress should be futher explored in other cell lines as well as primary cells.

These data provide supporting evidence for a mechanistic link between environmental oxidative stress and DNA methylation. We propose that oxidative stress can influence DNMT1 activity, causing longer term changes in cytosine methylation patterns. Although it is accepted that environmental factors can influence the epigenome, few studies have investigated the pathways through which this can occur and the effect in subsequent cell generations. These data demonstrate that exposure to oxidative stress during cellular replication corresponds with substantial decreases in DNA methylation, and while the majority of these changes are subsequently corrected, a smaller number of sites remain demethylated. Furthermore, exposure to H_2_O_2_ resulted in increased variability of methylation in a site-specific manner. This is of great interest for cancer biology where metabolic reprogramming and the tumour microenvironment combine to create a milieu of oxidative stress that can promote altered epigenetic profiles and modify cell behaviour of both neoplastic and normal cells.

## Conclusions

The results of this research indicate that the human Jurkat T-lymphoma epigenome is susceptible to sustained changes in DNA methylation after exposure to oxidative stress during cellular replication. These studies need to be expanded to other proliferating cells. H_2_O_2_ exposure was associated with genome-wide decreases in DNA methylation, which were largely corrected during subsequent cycles of cell division. The magnitude of the observed changes generally decrease with time, however, a subset of changes that displayed a relatively small initial effect size at 4 h demonstrated an increased effect size at 72 h. These results suggest that oxidant-induced changes in DNA methylation can be inherited by subsequent cell generations. Furthermore, we show that treatment with H_2_O_2_ affects variability of methylation in a global and site-specific manner.

## Methods

### Cell culture reagents

All experiments were performed using E6.1 Jurkat CD4 + /CD8- T-cell lymphoma cell line. Cells were cultured in RPMI 1640 + 2 mM glutamine + 10% fetal bovine serum (FBS) and 1% penicillin/streptomycin (all from Gibco, Life Technologies), and incubated in 5% CO_2_ at 37 °C. Hydrogen peroxide was diluted into PBS and its concentration determined spectrophotometrically (ε_240_ = 43.6 M^−1^ cm^−1^).

### Cell synchronization

Cellular replication of E6.1 Jurkat cells was synchronized using a single thymidine block protocol to arrest cells between late G_1_ and early S-phase. Briefly, cells (1 million cells/ml) were treated with thymidine (2′-deoxythymidine, Sigma) at a final concentration of 1 mM and incubated for 18 h. To promote release into the S-phase, the block was alleviated by two media changes and addition of cytidine (2′-deoxycytidine, Sigma) to a final concentration of 50 µM. Cell cycle progression was assessed by flow cytometry every two hours over a 12-h period by analysing cellular DNA content following cell fixing in ethanol and staining with propidium iodide (PI) (Sigma). This approach was used to detect cells in each of the three major phases of the cycle (G_0_/G_1_, S and G_2_/M), and also allowed for detection of apoptotic cells with minimal DNA content.

### Cell culture procedure

Each of four replicates originated from frozen cells of differing passage lineages, and the following procedure was performed for each replicate, on independent days. Cellular synchronization was performed on 200 × 10^6^ cells in 200 mL as described above. Immediately after release, the cells were split into two equal volumes at 0.5 × 10^6^/ mL for treatment and control samples. At two hours post-release, cells in media were treated with H_2_O_2_ at a final concentration of 10 µM. This was repeated one hour later, meaning the cells received two H_2_O_2_ bolus doses totaling 20 µM. Cells were split to a concentration of 250,000 cells/ mL after 24 h. Samples containing approximately five million cells, were collected at four hours and 72-h post-release for DNA extraction. Flow cytometry for cell viability (using PI), cell cycle analyses, and live cell counts were performed at pre-block, pre-release, and 4, 24, 48 and 72-h post-release time points.

### Cell viability, proliferation and cell cycle progression

The percentage of viable cells at each major time point was assessed by the exclusion of PI. Cell cycle analyses were performed by fixation with ethanol followed by incubation with PI for 30 min. Cell proliferation was observed by labelling cells with carboxyfluorescein diacetate succinimidyl ester (CFSE; Invitrogen, New Zealand), as described previously [77].

### DNA extraction and bisulfite conversion

DNA was extracted from cell samples using a GeneJet DNA extraction kit (ThermoFisher Scientific, USA) according to the manufacturer’s instructions. Sodium bisulfite treatment of genomic DNA was performed on 1000 ng of DNA using the Zymo Research EZ DNA methylation kit, according to the manufacturer’s recommended protocol for use on the Illumina Infinium MethylationEPIC 850K array. Cells harvested at 4 h and 72 h after cell cycle release were assessed for DNA methylation profiles using the Illumina methylationEPIC bead chip array. Methylated and non-methylated values were determined using the minfi pipeline, within R. To control for DNA methylation changes that occur naturally during cellular replication, the block-release protocol was performed on all cells together.

### Bioinformatics analyses

DNA methylation profiles of the control samples were subtracted from the treatment samples. Data analyses were performed using the Minfi and Limma Bioconductor software packages within the R statistical program (www.R-project.org). All packages, programs and pipelines used in these analyses are freely available, and the workflow was based upon published scripts [78]. Any additional scripts are available from the authors on request. All samples passed quality control together using Subset-quantile Within Array Normalization (SWAN) [79] and variance-stabilizing transformation [80, 81]. Probes containing detection *p*-values > 0.05 for 1% or more samples, were excluded from further analysis. Because the cell samples were of the same sex, all chromosomes were retained, however, probes identified as having polymorphic hybridizing potential and homology to common SNPs [82] were removed. The final data frame contained 809,911 probes available for analysis. Multidimensional scaling of the top 1000 methylation values was performed using pairwise distance method for gene selection, and normalized, filtered methylation values. Hierarchical clustering was performed on a “minkowski” distance matrix calculated using the β-values for all probes, regardless of significance [83].

### Average relative DNA methylation changes

Average relative DNA methylation changes were calculated using mean β-values from all probes for each sample in the four groups. The absolute mean differences between control and treatment groups at each time point were calculated using paired t-tests.

### Differential variably

Differentially variable positions (DVPs) were identified using the DiffVar algorithm [84] within the missMethyl package [85]. This analysis used a similar linear model as below, however, biological replicates were incorporated into the statistical design as covariates.

### Identification of differentially methylated CpGs

The methylation status of each probe was calculated using normalized probe signals represented as methylation values (M-values) and β-values. M-values were generated within Minfi as the log_2_ ratio of the signal intensities of methylated probe divided by the unmethylated probe. Unless stated otherwise, statistical analyses were performed using the M-values. β-values (average DNA methylation level for each probe) were used for data visualization and range from 0 (unmethylated) to 1 (methylated). β-values were generated by dividing the methylated probe signal with the sum of the methylated and unmethylated probe signals [86]. Normalized β-values and M-values were manually assessed for fit (Additional file 1: Figure S1).

Differentially methylated positions which correlated with treatment were identified using a linear regression model within the Limma package, with adjustment for multiple testing. This was calculated by comparing the methylation measurements of the control samples with the treatment samples, for each of the two time points (4-h post-release, and 72-h post-release). Samples that originated from the same cell passage were treated as a biological replicate and the correlation coefficient between these samples was incorporated into the statistical design using Limma’s duppcorr function. The top most significant, differentially methylated CpG positions were identified by log fold change (logFC) weighted functions using Limma’s topTreat algorithm. Adjustment for multiple correction was performed using the “Benjamini, Hochberg” method within Limma and Quantile–Quantile plots for the linear regression model were assessed at each time point using the observed against expected –log_10_(*p*-value) (Additional file 1: Fig. S2). The observed data show a minor inflation of *p*-values smaller than expected by chance. Therefore, we substantially reduced our criteria for significance by excluding probes that displayed less than a 10% mean difference in methylation between treatment and control samples. This also removed significant changes observed at sites that displayed methylation values less than 10% or greater than 90%, as changes within these ranges are less likely to be of biological relevance.

Pathway analysis was performed by comparison with the Kyoto Encyclopedia of Genes and Genomes (KEGG) [87] and gene ontology databases (GO) [88] using the missMethyl R package [85], with correction for probe bias.

### Differentially methylated regions

Differentially methylated regions were interrogated within the Minfi package using the statistical package DMRcate [89]. A methylation differential cutoff of 10 was used, and unless stated otherwise significance was determined using a false discovery rate (FDR) of 0.05 in conjunction with a *p-*value cutoff of 0.05.

## Supplementary Information

**Additional file 1.** Supplementary tables and figures.

## Data Availability

All data generated or analysed during this study are included in this published article and its Additional files. Original scripts used for data analysis are available from the corresponding authors upon request.

## Abbreviations

DNMT: DNA methyltransferase
SAM: S-Adenosyl-methionine
CFSE: Carboxyfluorescein diacetate succinimidyl ester
PCA: Principal component analysis
DVPs: Differential variability at individual CpG positions
DMRs: Differentially methylated regions
GO: Gene ontology database
KEGG: Kyoto Encyclopedia of Genes and Genomes
