# Supplementary material for: Genome-wide impact of hydrogen peroxide on maintenance DNA methylation in replicating cells
Source: Epigenetics Chromatin. 2021 Mar 24;14:17. doi: 10.1186/s13072-021-00388-6 (PMC7992848; doi:10.1186/s13072-021-00388-6)
Supplement: Supplementary file 1 — Additional file 1. Supplementary tables and figures. [file 13072_2021_388_MOESM1_ESM.docx]

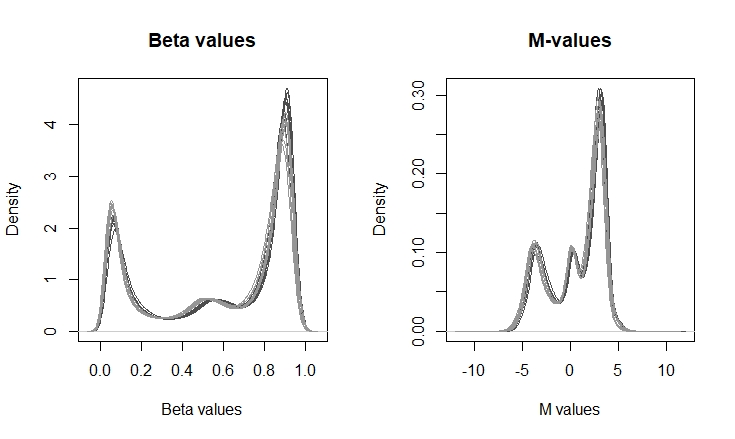


**Supplementary Figure 1. Assessment of normalized β -values and M-values.** Control samples are represented in dark grey and treatment samples are represented in light grey. The bump observed at ~50% methylation is characteristic of Jurkat cells, which have a highly duplicated genome.


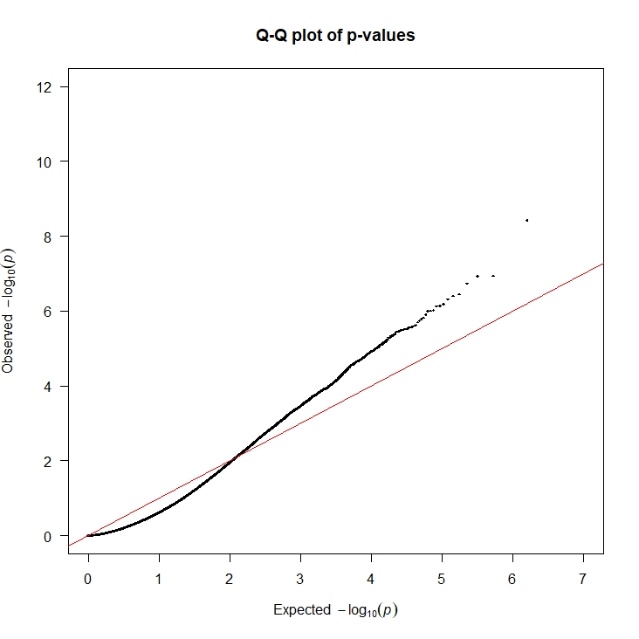

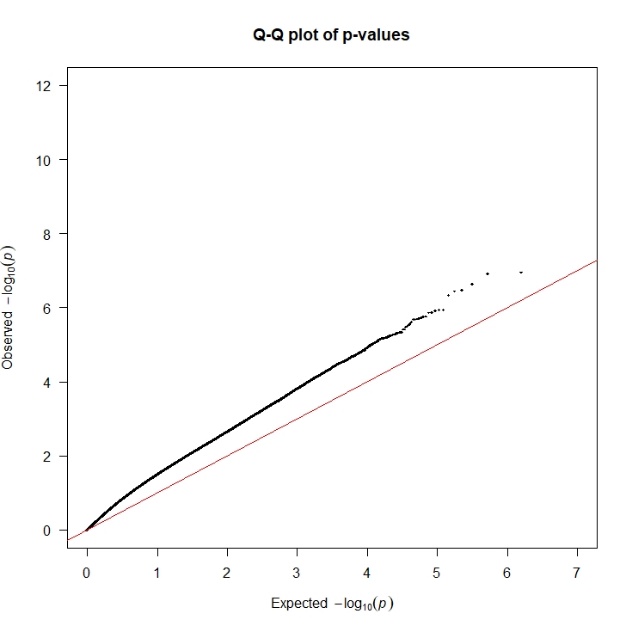


**Supplementary Figure 2. Quantile-quantile plot on expected –log(p-values) (x-axis) vs observed –log(p-values) ( y-axis).** Under the null distribution, of no significant associations, all points would be expected to lie on the red line at y = x. A) 4 hour time-point B) 72 hour time-point.

Supplementary Table 1. Top differentially methylated probes that display a (M-value) logFC greater than log_2_(1.2), as identified by Limma’s “topTreat” function and ordered by magnitude of log_2_FC. Probes were excluded if there was less than a 10% difference between treatment and control, and also if the control sample had a mean methylation value less than 10% or greater than 90%, which was assessed using beta values.

| Probe ID | Log_2_FC | t | adj.*p*.val | Chromosome | Location | Gene |
| --- | --- | --- | --- | --- | --- | --- |
| cg21150892 | 2.94 | 5.75 | 0.04 | 1 | 183846009 | RGL1 |
| cg26890348 | 2.48 | 6.41 | 0.03 | 13 | 52637074 |  |
| cg24024260 | -2.29 | -7.47 | 0.01 | 2 | 182174340 | LOC101927156 |
| cg05047191 | -2.23 | -5.59 | 0.04 | 12 | 15304108 | RERG |
| cg00192946 | -2.19 | -7.55 | 0.01 | X | 32430274 | DMD |
| cg21426441 | -2.19 | -5.56 | 0.04 | X | 17260259 |  |
| cg14954143 | -2.13 | -6.56 | 0.02 | 3 | 162071211 |  |
| cg02187176 | 2.1 | 5.8 | 0.04 | 13 | 96367153 | DNAJC3 |
| cg23727674 | -2.09 | -5.94 | 0.03 | 2 | 148602993 | ACVR2A |
| cg25969765 | -2.07 | -6.01 | 0.03 | 18 | 58350853 |  |
| cg11372135 | -2.05 | -7.3 | 0.01 | 10 | 18629284 | CACNB2 |
| ch.X.16400295F | -2.04 | -6.51 | 0.02 | X | 16490374 |  |
| cg03012782 | -1.87 | -6.16 | 0.03 | 3 | 112250543 |  |
| cg06780892 | -1.87 | -5.71 | 0.04 | 7 | 20638177 |  |
| cg15074165 | -1.85 | -5.9 | 0.03 | X | 71792745 | HDAC8 |
| cg09333215 | -1.84 | -7.71 | 0.01 | 1 | 88475866 |  |
| cg01492091 | -1.81 | -6.78 | 0.02 | 7 | 31460998 |  |
| cg08892255 | -1.8 | -6.1 | 0.03 | 20 | 21928217 |  |
| cg07042371 | -1.77 | -6.59 | 0.02 | 5 | 54468233 | MIR449C |
| cg01769501 | -1.77 | -5.8 | 0.04 | 4 | 76912251 | SDAD1 |
| cg17476352 | -1.74 | -6.22 | 0.03 | 8 | 115103191 |  |
| cg23479323 | -1.72 | -5.97 | 0.03 | X | 106061616 | TBC1D8B |
| cg14292200 | -1.72 | -5.78 | 0.04 | 5 | 172682189 |  |
| cg22205811 | -1.72 | -5.67 | 0.04 | 5 | 105737302 |  |
| cg09814198 | -1.7 | -6.2 | 0.03 | 8 | 64751976 |  |
| cg22991142 | -1.7 | -6.18 | 0.03 | 20 | 9640155 | PAK7 |
| cg01703245 | -1.69 | -5.83 | 0.03 | X | 150566421 | VMA21 |
| cg26746986 | -1.69 | -5.45 | 0.05 | 10 | 118177499 |  |
| ch.3.2279603R | -1.68 | -6.62 | 0.02 | 3 | 116239886 |  |
| cg14710666 | -1.67 | -5.87 | 0.03 | 2 | 160803214 | PLA2R1 |
| cg10109286 | -1.65 | -6.46 | 0.03 | 4 | 112724069 |  |
| cg08894816 | -1.65 | -6.11 | 0.03 | 7 | 141647337 | CLEC5A |
| cg22128954 | -1.65 | -6.05 | 0.03 | 1 | 165087522 |  |
| cg04201788 | -1.63 | -6.16 | 0.03 | 5 | 39570723 |  |
| cg21195920 | -1.62 | -10.6 | 0.001 | 15 | 90944458 | IQGAP1 |
| cg18949056 | -1.62 | -6.84 | 0.02 | 10 | 97317243 | SORBS1 |
| cg16466107 | -1.61 | -5.55 | 0.04 | 12 | 16430439 | SLC15A5 |
| cg04254259 | -1.6 | -7.32 | 0.01 | 4 | 68996281 | TMPRSS11F |
| cg04362419 | -1.6 | -6.71 | 0.02 | 3 | 138297086 | CEP70 |
| cg14934522 | -1.59 | -6.57 | 0.02 | 4 | 77618676 | SHROOM3 |
| cg06812574 | -1.56 | -6.7 | 0.02 | 10 | 18331664 | SLC39A12 |
| cg27559254 | -1.56 | -5.84 | 0.03 | 7 | 115316863 |  |
| cg00312625 | -1.54 | -5.58 | 0.04 | 8 | 112439876 |  |
| cg10792660 | -1.52 | -6.87 | 0.02 | 4 | 125369980 |  |
| cg05930091 | -1.52 | -5.91 | 0.03 | 8 | 87077897 | PSKH2 |
| cg06403830 | -1.5 | -7.05 | 0.02 | 2 | 202279205 | TRAK2 |
| cg03964941 | -1.49 | -5.75 | 0.04 | 6 | 26273127 | HIST1H2BI |
| cg21028981 | -1.49 | -5.6 | 0.04 | 3 | 192465728 |  |
| cg20487384 | -1.47 | -6.65 | 0.02 | 4 | 90472345 |  |
| cg02830467 | -1.46 | -6.38 | 0.03 | 6 | 153018721 | MYCT1 |
| cg06723404 | -1.46 | -5.48 | 0.05 | 2 | 44589978 | PREPL;CAMKMT |
| cg05577994 | -1.45 | -6.8 | 0.02 | 10 | 119254968 | EMX2OS |
| cg02889647 | -1.45 | -6.16 | 0.03 | 1 | 152879586 | IVL |
| cg06000491 | -1.45 | -6.05 | 0.03 | 2 | 34327576 | LINC01317 |
| cg03550208 | -1.44 | -6.79 | 0.02 | 5 | 6582193 | LOC255167 |
| cg03719252 | -1.43 | -6.76 | 0.02 | 6 | 140529431 |  |
| cg24486736 | -1.43 | -5.82 | 0.03 | 13 | 43451777 |  |
| cg06635240 | -1.43 | -5.77 | 0.04 | X | 33359128 | DMD |
| cg05519451 | -1.41 | -5.84 | 0.03 | 4 | 41727699 |  |
| cg12598924 | -1.4 | -6.12 | 0.03 | X | 99893099 | TSPAN6 |
| cg20700869 | -1.39 | -7.19 | 0.02 | 8 | 141775220 | PTK2 |
| cg07872987 | -1.38 | -5.59 | 0.04 | 3 | 111260980 | CD96 |
| cg03144446 | -1.38 | -5.5 | 0.05 | 7 | 20366667 |  |
| ch.20.750973F | -1.37 | -6.37 | 0.03 | 20 | 35650572 | RBL1 |
| cg06369657 | -1.37 | -5.97 | 0.03 | 7 | 137311110 | DGKI |
| cg00079551 | -1.37 | -5.97 | 0.03 | 2 | 40001222 | THUMPD2 |
| cg19049964 | -1.35 | -5.66 | 0.04 | 5 | 2744422 |  |
| cg00196810 | -1.34 | -6.67 | 0.02 | 3 | 53925267 | SELK |
| cg10563213 | -1.34 | -6.26 | 0.03 | 5 | 167147173 | TENM2 |
| cg09229115 | -1.33 | -6.07 | 0.03 | 13 | 91741887 | LINC00380 |
| cg00187128 | -1.32 | -5.91 | 0.03 | 4 | 128608153 | INTU |
| cg18447751 | -1.32 | -5.82 | 0.03 | 21 | 17894815 | MIR99AHG |
| cg10540928 | -1.31 | -5.48 | 0.05 | X | 67625174 | OPHN1 |
| cg19924480 | -1.3 | -5.87 | 0.03 | X | 36594674 |  |
| cg00002154 | -1.27 | -6.22 | 0.03 | 4 | 59941655 |  |
| cg14127414 | -1.26 | -5.98 | 0.03 | 1 | 220998978 |  |
| cg13057239 | -1.26 | -5.98 | 0.03 | 4 | 176780685 | GPM6A |
| cg01449425 | -1.25 | -5.49 | 0.05 | 3 | 181957089 |  |
| cg00440859 | -1.23 | -6.77 | 0.02 | X | 115028898 | DANT2 |
| cg18711270 | -1.23 | -5.82 | 0.03 | 8 | 17558318 | MTUS1 |
| cg22871229 | -1.22 | -5.75 | 0.04 | 2 | 68690716 | FBXO48 |
| cg11544398 | -1.19 | -7.25 | 0.02 | 18 | 25955245 |  |
| cg17946713 | -1.17 | -5.73 | 0.04 | 5 | 14108721 |  |
| cg21792583 | -1.11 | -6.31 | 0.03 | 15 | 51616613 | CYP19A1 |
| cg06507137 | -1.11 | -5.94 | 0.03 | 5 | 27141272 |  |
| cg07761822 | -1.03 | -5.97 | 0.03 | 4 | 26323722 | RBPJ |
| cg20880031 | -1.02 | -5.52 | 0.04 | 7 | 103630989 | RELN |
| cg20576896 | -1.01 | -7.34 | 0.01 | 12 | 64521378 | SRGAP1 |
| cg05732191 | -1 | -6.55 | 0.02 | 6 | 28191910 | ZSCAN9 |
| cg11789612 | -0.91 | -5.45 | 0.05 | 15 | 41794412 | ITPKA |

**Log_2_FC, Log2 fold change of M values**

**Adj.p-val., adjusted *p*-value**

Supplementary Table 2. Top 100 differentially methylated probes, ordered ordered by magnitude of log_2_FC for the 72 hour time point, assessed using Limma’s “topTreat function, with a logFC>1.

| Probe ID | Log_2_FC | adj.p.Val | Gene | Chromosome | Location |
| --- | --- | --- | --- | --- | --- |
| cg21998794 | -1.72 | 0.04 | ELP4;IMMP1L | 11 | 31530551 |
| cg02947434 | -1.72 | 0.05 |  | 12 | 33728505 |
| cg26627970 | -1.64 | 0.04 | KHDRBS2 | 6 | 62993589 |
| cg13484357 | -1.6 | 0.05 | SLC30A8 | 8 | 118125096 |
| cg10578504 | -1.54 | 0.04 | SLC30A8 | 8 | 118145984 |
| cg06328725 | -1.5 | 0.05 | PACRG | 6 | 163171959 |
| cg05620710 | -1.48 | 0.05 |  | 5 | 140010064 |
| cg05403127 | -1.43 | 0.06 | LINC00703 | 10 | 4425587 |
| cg05655613 | -1.42 | 0.02 |  | 11 | 105101601 |
| cg08223309 | -1.41 | 0.04 |  | 4 | 14390632 |
| cg01909661 | -1.4 | 0.05 | PTCHD1-AS | X | 22446176 |
| cg11932468 | -1.38 | 0.05 | TRPA1;MSC-AS1 | 8 | 72954594 |
| cg19281347 | -1.38 | 0.05 |  | 2 | 83883578 |
| cg02153561 | -1.37 | 0.06 |  | 18 | 70077275 |
| cg06951009 | -1.35 | 0.06 |  | 4 | 12547176 |
| cg22181263 | -1.31 | 0.02 |  | 6 | 63922844 |
| cg21898358 | -1.31 | 0.06 | LILRA | 19 | 54805375 |
| cg18363176 | -1.26 | 0.05 | FLI1 | 11 | 128606110 |
| cg05577994 | -1.25 | 0.04 | EMX2OS | 10 | 119254968 |
| cg23537032 | -1.25 | 0.06 |  | 6 | 24165998 |
| cg18998172 | -1.25 | 0.06 | SPOCK1 | 5 | 136340207 |
| cg11424548 | -1.22 | 0.04 |  | 10 | 120000847 |
| cg13871484 | 1.21 | 0.06 | UHMK1 | 1 | 162466506 |
| ch.20.848555F | -1.21 | 0.06 | TOP1 | 20 | 39748531 |
| cg08149869 | -1.19 | 0.05 |  | 21 | 24264513 |
| cg14699663 | -1.18 | 0.04 | GPR1 | 2 | 207082644 |
| cg22383292 | -1.18 | 0.04 | THSD7A | 7 | 11412105 |
| cg22999327 | -1.18 | 0.05 | PDE3A | 12 | 20700997 |
| cg22223530 | -1.18 | 0.06 | ST8SIA2 | 15 | 92977495 |
| cg24707035 | -1.17 | 0.04 | IGF1 | 12 | 102868087 |
| cg00963748 | -1.17 | 0.05 | LRRC9 | 14 | 60385180 |
| cg16627854 | -1.17 | 0.05 |  | 6 | 126848448 |
| cg13816833 | -1.17 | 0.05 |  | 7 | 79249781 |
| cg00635343 | -1.16 | 0.06 | LOC642597 | 18 | 5193969 |
| ch.2.3268406F | -1.15 | 0.02 | BAZ2B | 2 | 160224706 |
| cg20097712 | -1.15 | 0.06 |  | 11 | 124172447 |
| cg14490945 | -1.14 | 0.02 | LINC00598 | 13 | 40996323 |
| cg00637744 | -1.14 | 0.06 | LINC01122 | 2 | 59239404 |
| cg27627737 | -1.14 | 0.06 |  | 6 | 82393976 |
| cg15737862 | -1.12 | 0.05 |  | 9 | 7234317 |
| cg02371397 | -1.11 | 0.05 | RYR3 | 15 | 33799380 |
| ch.5.158370R | -1.11 | 0.06 |  | 5 | 4321970 |
| cg15211032 | 1.11 | 0.06 | AP1S2 | X | 15863925 |
| cg25479340 | 1.1 | 0.04 | LOC105370306 | 13 | 88794067 |
| cg00445187 | -1.1 | 0.06 | PITX2 | 4 | 111564206 |
| cg04489316 | 1.1 | 0.06 | HDAC8 | X | 71687141 |
| cg18216587 | -1.09 | 0.04 | CNTNAP2 | 7 | 146019420 |
| cg17347305 | -1.09 | 0.04 |  | 5 | 102803554 |
| cg02559410 | 1.09 | 0.05 | MKL2 | 16 | 14169732 |
| cg01649601 | -1.09 | 0.06 |  | 8 | 40201327 |
| cg17835169 | -1.08 | 0.05 | PDE6A | 5 | 149281308 |
| cg14981883 | -1.07 | 0.05 |  | 10 | 8084742 |
| cg22962811 | -1.06 | 0.04 |  | 4 | 181868775 |
| cg23609811 | -1.06 | 0.05 | HIST1H2BJ;HIST1H2AG | 6 | 27100717 |
| cg16628093 | -1.05 | 0.06 | CALCRL | 2 | 188274544 |
| cg27374159 | -1.04 | 0.04 | UGT8 | 4 | 115580778 |
| cg21261985 | -1.03 | 0.05 |  | 7 | 93824706 |
| cg07778635 | -1.03 | 0.06 | IQUB | 7 | 123095978 |
| cg05490029 | -1.01 | 0.05 | IL7 | 8 | 79719015 |
| cg12068600 | -1.01 | 0.06 |  | 1 | 118353410 |
| cg21085188 | -1.01 | 0.06 |  | 6 | 47289489 |
| cg21682137 | -1.01 | 0.06 | PEG3;ZIM2 | 19 | 57336525 |
| cg09837911 | -1.01 | 0.06 |  | 17 | 75931245 |
| cg15864256 | -1 | 0.05 |  | 12 | 91755828 |
| cg21268262 | -1 | 0.06 |  | 11 | 87431020 |
| cg24254672 | -0.99 | 0.05 |  | 12 | 90479678 |
| cg20206019 | -0.98 | 0.06 | DSCAM | 21 | 41975426 |
| cg20936586 | -0.98 | 0.06 | LYPD6B | 2 | 149998441 |
| cg15577048 | -0.97 | 0.05 |  | 14 | 43585896 |
| cg18830373 | -0.96 | 0.06 | MIR548F5;NBEA | 13 | 36110868 |
| cg14412160 | -0.95 | 0.05 | BMP3 | 4 | 81957210 |
| cg17052410 | -0.95 | 0.06 |  | 11 | 24477391 |
| cg02577240 | -0.95 | 0.06 | SLC8A1 | 2 | 40739938 |
| cg06924602 | -0.95 | 0.06 | SBSN | 19 | 36017061 |
| cg04806664 | -0.95 | 0.06 |  | 14 | 97135129 |
| cg11228403 | -0.94 | 0.05 | LMNTD1 | 12 | 25710967 |
| cg27659862 | -0.94 | 0.06 | MYO1D | 17 | 30882177 |
| cg03736057 | -0.94 | 0.06 |  | 4 | 187647197 |
| cg06096373 | -0.93 | 0.06 |  | 17 | 13339885 |
| cg18822544 | -0.92 | 0.05 | KRTAP26-1 | 21 | 31692465 |
| cg23128005 | -0.91 | 0.05 | CNTN1 | 12 | 41085727 |
| cg11892366 | -0.91 | 0.05 |  | 5 | 25090986 |
| cg07999668 | -0.9 | 0.06 | AICDA | 12 | 8765336 |
| cg10273096 | -0.89 | 0.06 | C9orf130 | 9 | 98637454 |
| cg00957901 | -0.88 | 0.06 | LIMCH1 | 4 | 41614842 |
| cg03367149 | -0.86 | 0.06 |  | 3 | 81304743 |
| cg02892281 | -0.84 | 0.06 | HSP90AB1 | 6 | 44215596 |
| cg22666688 | -0.83 | 0.06 |  | X | 111755938 |
| cg05994561 | -0.79 | 0.05 |  | 7 | 119783665 |
| cg13576344 | -0.79 | 0.05 |  | 2 | 217764227 |
| cg25111228 | -0.78 | 0.06 | NPAS3 | 14 | 34262104 |
| cg00362062 | -0.73 | 0.06 | KCNQ3 | 8 | 133332014 |
| cg04348283 | -0.72 | 0.06 | TRIO | 5 | 14387294 |
| cg24747763 | -0.7 | 0.05 | CRISP3 | 6 | 49705127 |
| cg17038667 | -0.66 | 0.05 | FGF3 | 11 | 69632883 |
| cg04124998 | -0.62 | 0.06 | AGMO | 7 | 15602436 |
| cg00345443 | -0.61 | 0.05 |  | 3 | 54121778 |
| cg14934481 | -0.6 | 0.06 |  | 7 | 19159452 |
| cg25329318 | -0.58 | 0.04 |  | 5 | 38253946 |

**Log_2_FC, Log2 fold change of M values**

**Adj.p-val., adjusted *p*-value**

Supplementary Table 3. Significant DMRs observed for the 4 hour time-point, obtained using the dmrcate algorithm within R.

| Chromosome | start | end | width | #cpgs | minfdr | maxbetafc | meanbetafc | overlapping.promoters |
| --- | --- | --- | --- | --- | --- | --- | --- | --- |
| chr4 | 38857850 | 38859770 | 1921 | 11 | 2.67E-20 | -0.12 | -0.05 | TLR6-201, TLR1-004, TLR6-002, TLR6-003 |
| chr6 | 25166749 | 25168331 | 1583 | 6 | 7.64E-16 | -0.10 | -0.05 | CMAHP-201, CMAHP-007 |
| chr11 | 19223517 | 19224670 | 1154 | 6 | 2.75E-13 | -0.09 | -0.07 | CSRP3-201 |
| chr7 | 144107418 | 144108266 | 849 | 7 | 9.36E-11 | -0.09 | -0.05 | NOBOX-002, NOBOX-001 |
| chr1 | 165393661 | 165395708 | 2048 | 8 | 2.86E-08 | -0.07 | -0.05 | RXRG-002 |
| chr4 | 41875193 | 41875875 | 683 | 6 | 7.93E-12 | -0.15 | -0.05 | NA |
| chr8 | 76450694 | 76452152 | 1459 | 5 | 1.31E-10 | -0.09 | -0.06 | HNF4G-001 |
| chr6 | 55190909 | 55192338 | 1430 | 7 | 6.16E-12 | -0.16 | -0.06 | GFRAL-001 |
| chr7 | 26896340 | 26897714 | 1375 | 7 | 4.52E-08 | -0.07 | -0.05 | SKAP2-002, SKAP2-004, SKAP2-005, SKAP2-201 |
| chr15 | 89178776 | 89179370 | 595 | 6 | 3.43E-09 | -0.06 | -0.05 | ISG20-010, ISG20-009 |
| chr6 | 15551063 | 15553155 | 2093 | 5 | 4.32E-09 | -0.07 | -0.05 | NA |
| chr4 | 105827067 | 105828553 | 1487 | 6 | 9.45E-08 | -0.07 | -0.05 | RP11-556I14.1-001, RP11-556I14.1-003 |
| chr3 | 73610745 | 73611772 | 1028 | 7 | 8.24E-09 | -0.09 | -0.05 | PDZRN3-004 |
| chr8 | 57033930 | 57034981 | 1052 | 6 | 1.86E-09 | -0.06 | -0.05 | SNORA3.1-201 |
| chr3 | 172693052 | 172694490 | 1439 | 5 | 4.98E-07 | -0.08 | -0.06 | snoU13.410-201 |
| chr13 | 41556139 | 41557552 | 1414 | 8 | 1.78E-10 | -0.08 | -0.05 | ELF1-201, ELF1-001 |
| chr16 | 77322331 | 77324732 | 2402 | 5 | 1.25E-06 | -0.08 | -0.06 | ADAMTS18-011 |
| chr6 | 72113322 | 72114693 | 1372 | 6 | 1.39E-11 | -0.13 | -0.05 | MIR30A-201 |
| chr5 | 149635090 | 149636432 | 1343 | 5 | 1.61E-07 | -0.08 | -0.06 | NA |
| chr7 | 47431717 | 47433140 | 1424 | 5 | 2.65E-07 | -0.08 | -0.05 | NA |
| chrX | 77154697 | 77155144 | 448 | 8 | 1.50E-07 | -0.08 | -0.05 | COX7B-002, COX7B-003, COX7B-001 |
| chr5 | 14108303 | 14108781 | 479 | 5 | 3.96E-14 | -0.17 | -0.06 | NA |
| chr8 | 124741531 | 124743500 | 1970 | 5 | 1.73E-06 | -0.08 | -0.06 | ANXA13-006, ANXA13-004, ANXA13-005, ANXA13-003 |
| chr18 | 76637503 | 76640314 | 2812 | 6 | 1.24E-06 | -0.12 | -0.06 | NA |
| chr20 | 60073683 | 60074520 | 838 | 7 | 4.77E-09 | -0.09 | -0.06 | NA |
| chr11 | 7847155 | 7848885 | 1731 | 5 | 1.83E-07 | -0.09 | -0.06 | OR5P3-001 |
| chr16 | 87249388 | 87250689 | 1302 | 6 | 7.90E-12 | -0.10 | -0.05 | RP11-899L11.3-001 |
| chr16 | 50701064 | 50702614 | 1551 | 5 | 2.58E-06 | -0.08 | -0.06 | RP11-401P9.5-001, RP11-401P9.5-002 |
| chr6 | 133035150 | 133035379 | 230 | 5 | 2.14E-07 | -0.12 | -0.06 | VNN1-001 |
| chr8 | 33866594 | 33867555 | 962 | 5 | 1.89E-08 | -0.09 | -0.05 | RP1-273G13.3-001, RP11-317N12.1-002, RP1-273G13.3-002,  RP11-317N12.1-001 |
| chr19 | 41495868 | 41496749 | 882 | 5 | 1.87E-07 | -0.08 | -0.05 | CYP2B6-001, CYP2B6-004 |
| chr15 | 27184766 | 27185988 | 1223 | 5 | 7.03E-10 | -0.09 | -0.05 | GABRB3-003 |
| chr9 | 14722242 | 14723496 | 1255 | 5 | 5.63E-06 | -0.07 | -0.05 | CER1-001 |
| chr9 | 138108675 | 138110289 | 1615 | 5 | 4.17E-07 | -0.09 | -0.05 | NA |
| chr4 | 171194243 | 171195014 | 772 | 5 | 6.53E-08 | -0.10 | -0.06 | RP11-789C1.1-001 |
| chr7 | 3995563 | 3997610 | 2048 | 5 | 1.56E-05 | -0.08 | -0.05 | NA |
| chr7 | 25702709 | 25703841 | 1133 | 5 | 4.89E-06 | -0.09 | -0.05 | AC003090.1-002, AC003090.1-001 |
| chr3 | 171488952 | 171490453 | 1502 | 5 | 1.29E-07 | -0.08 | -0.06 | PLD1-010, PLD1-009 |
| chr16 | 28009836 | 28011265 | 1430 | 5 | 8.31E-06 | -0.10 | -0.05 | RNU6-1241P-201 |
| chr2 | 199179295 | 199180751 | 1457 | 6 | 3.17E-09 | -0.08 | -0.05 | AC005235.1-002 |
| chr2 | 79386720 | 79388347 | 1628 | 5 | 2.32E-24 | -0.10 | -0.06 | REG3A-002, REG3A-201, REG3A-001, AC011754.1-001,  REG3A-004, REG3A-003 |
| chrX | 99847172 | 99848987 | 1816 | 5 | 9.34E-09 | -0.19 | -0.06 | TNMD-002 |
| chr9 | 74675332 | 74676496 | 1165 | 5 | 6.48E-13 | -0.09 | -0.06 | C9orf57-001 |
| chr17 | 35302132 | 35303488 | 1357 | 5 | 3.46E-05 | -0.09 | -0.05 | RP11-445F12.2-001 |
| chr19 | 29284229 | 29285119 | 891 | 5 | 8.48E-06 | -0.08 | -0.05 | CTC-478M6.1-001 |
| chr5 | 9630560 | 9631798 | 1239 | 5 | 7.71E-14 | -0.07 | -0.05 | TAS2R1-001 |
| chr8 | 113654569 | 113656015 | 1447 | 5 | 5.78E-10 | -0.09 | -0.05 | MIR2053-201 |
| chr7 | 80252042 | 80254266 | 2225 | 5 | 3.19E-10 | -0.10 | -0.05 | CD36-005, CD36-007, CD36-004, CD36-010 |
| chr4 | 171664902 | 171665510 | 609 | 5 | 0.000176654 | -0.14 | -0.06 | RP11-322J23.1-001 |
| chr6 | 143485183 | 143486700 | 1518 | 5 | 6.86E-10 | -0.09 | -0.05 | AIG1-008 |
| chr2 | 168103475 | 168105326 | 1852 | 8 | 1.15E-06 | -0.10 | -0.06 | NA |
| chr11 | 85438773 | 85439999 | 1227 | 5 | 8.65E-07 | -0.12 | -0.06 | SYTL2-010, SYTL2-201, SYTL2-009, SYTL2-008 |
| chr6 | 167737937 | 167738592 | 656 | 5 | 1.06E-06 | -0.08 | -0.05 | TTLL2-001, TTLL2-003, TTLL2-002 |
| chr5 | 140772023 | 140772512 | 490 | 8 | 1.53E-12 | -0.13 | -0.06 | PCDHGA8-001 |
| chr12 | 91505265 | 91506893 | 1629 | 6 | 3.02E-08 | -0.11 | -0.05 | LUM-001, LUM-003, LUM-002 |
| chrX | 17091579 | 17092517 | 939 | 5 | 3.25E-07 | -0.07 | -0.05 | REPS2-004 |
| chr15 | 92715189 | 92716386 | 1198 | 5 | 2.79E-05 | -0.15 | -0.06 | RP11-24J19.1-001, SLCO3A1-015 |
| chr21 | 15579335 | 15580581 | 1247 | 5 | 2.32E-08 | -0.11 | -0.05 | LIPI-001 |
| chr11 | 5067039 | 5068157 | 1119 | 5 | 7.85E-06 | -0.10 | -0.06 | OR52J3-001 |
| chr20 | 21927705 | 21928217 | 513 | 5 | 3.05E-11 | -0.15 | -0.05 | RP5-1185K9.1-001 |
| chr2 | 37543459 | 37544288 | 830 | 5 | 3.06E-06 | 0.09 | 0.05 | PRKD3-201, PRKD3-001, PRKD3-005, PRKD3-002 |
| chr1 | 109727962 | 109730501 | 2540 | 6 | 0.006514171 | -0.15 | -0.05 | KIAA1324-001, KIAA1324-003 |
| chr17 | 80880759 | 80882296 | 1538 | 5 | 2.11E-05 | -0.23 | -0.07 | TBCD-021 |
| chrX | 32866249 | 32867997 | 1749 | 5 | 3.34E-06 | -0.09 | -0.05 | DMD-009, snoU13.321-201 |
| chr6 | 25407220 | 25408223 | 1004 | 5 | 2.37E-06 | -0.16 | -0.06 | AL160037.1-201 |
| chr17 | 77478528 | 77478927 | 400 | 6 | 0.000188831 | -0.16 | -0.05 | RBFOX3-201, RBFOX3-002 |
| chrX | 57617561 | 57618891 | 1331 | 5 | 0.000223801 | -0.12 | -0.05 | ZXDB-001 |
| chr2 | 148253144 | 148254087 | 944 | 6 | 0.000958231 | -0.22 | -0.05 | RNU6-715P-201 |
| chr6 | 125420338 | 125421530 | 1193 | 6 | 0.001437759 | -0.25 | -0.06 | NA |
| chr1 | 163172649 | 163173756 | 1108 | 5 | 7.95E-07 | -0.16 | -0.05 | RGS5-001, RGS5-005, RGS5-004, RGS5-006, RGS5-007 |

Supplementary Table 4. Significant DMRs observed for the72 hour time-point, obtained using the dmrcate algorithm within R.

| Chromosome | start | end | width | no.cpgs | minfdr | maxbetafc | meanbetafc | Overlapping promoters |
| --- | --- | --- | --- | --- | --- | --- | --- | --- |
| chr6 | 132910681 | 132911747 | 1067 | 5 | 3.27E-07 | -0.10 | -0.06 | TAAR5-001 |
| chr3 | 172693052 | 172694490 | 1439 | 5 | 8.44E-05 | -0.10 | -0.05 | snoU13.410-201 |
| chr11 | 4718962 | 4720347 | 1386 | 5 | 5.76E-06 | -0.12 | -0.05 | OR51E2-001, OR51E2-002 |
| chr4 | 171664902 | 171665510 | 609 | 5 | 0.01246 | -0.12 | -0.05 | RP11-322J23.1-001 |
| chr6 | 49704803 | 49706545 | 1743 | 5 | 4.61E-08 | -0.12 | -0.05 | CRISP3-201 |

Supplementary Table 5. Results from KEGG and Go pathway analysis containing top hits with an FDR < 0.2

|  | Ontology* | Function | N | DM ** | FDR |
| --- | --- | --- | --- | --- | --- |
| GO:0030054 | CC | cell junction | 531 | 386 | 0.10 |
| GO:0007156 | BP | homophilic cell adhesion via plasma membrane adhesion molecules | 129 | 99 | 0.24 |
| GO:0000122 | BP | negative regulation of transcription by RNA polymerase II | 707 | 488 | 0.27 |
| GO:0001501 | BP | skeletal system development | 120 | 93 | 0.27 |
| GO:0005509 | MF | calcium ion binding | 631 | 430 | 0.27 |
| GO:0005516 | MF | calmodulin binding | 168 | 132 | 0.27 |
| GO:0010975 | BP | regulation of neuron projection development | 22 | 22 | 0.27 |
| GO:0045211 | CC | postsynaptic membrane | 174 | 136 | 0.27 |
| GO:0098978 | CC | glutamatergic synapse | 346 | 258 | 0.27 |
| KEGG:hsa05033 |  | Nicotine addiction | 40 | 34.5 | 0.20 |
| KEGG:hsa05202 |  | Transcriptional misregulation in cancer | 186 | 132 | 0.20 |

* BP - biological process, CC - cellular component, MF - molecular function.

**number of genes that were differentially methylated
